# Contributions of injury deaths to changes in life expectancy and disparity: A comparative analysis of G7 countries over two decades

**DOI:** 10.1186/s12963-025-00409-6

**Published:** 2025-08-04

**Authors:** Firoozeh Bairami, Mohammad Hajizadeh, Ali Kiadaliri

**Affiliations:** 1 School of Health Administration, Dalhousie University, Halifax, Canada; 2 Department of Clinical Sciences Lund, Orthopaedics, Lund University, Lund, Remissgatan 4, Wigerthuset, SE-221 85 Sweden; 3 Division of Insurance Medicine, Department of Clinical Neuroscience, Karolinska Institute, Stockholm, Sweden

**Keywords:** Life expectancy, Life disparity, Avoidable causes of death, Injury, G7 countries

## Abstract

**Background:**

Despite the high level of economic development in the Group of Seven (G7) countries, injury deaths remain a public health concern in these countries. This paper examines the contribution of injury deaths to changes in life expectancy (LE) and life disparity (LD) in the G7 countries.

**Methods:**

We used annual data from the WHO mortality database to compute LE and LD during 2001-03 and 2017-19. The contributions of injury deaths to LE and LD changes for each sex were decomposed by age and cause using a continuous-change model.

**Results:**

Across the G7 countries combined, LE (LD) increased by 2.12 (0.25) and 2.73 (0.16) years for females and males, respectively. While most injury-related deaths contributed to increases in LE and decreases in LD, these gains were offset by negative contributions of unintentional poisoning, resulting in an overall negligible net contributions of injury deaths to changes in LE/LD across the G7 countries combined. The country-specific patterns revealed notable variations. Positive contributions of injury-related causes to changes in LE were more prominent in France (+ 0.38/+0.64 years for females/males), while negative contributions were most evident in the USA (-0.23/-0.42 years for females/males). Transport accidents emerged as the leading contributors to improvements in both LE and LD among both sexes in all countries, with more pronounced effects in males. In contrast, unintentional poisoning had a substantial negative impact, particularly among younger populations in the USA, UK, and Canada.

**Conclusion:**

Injury deaths made negligible contributions to overall changes in LE and LD across the G7 countries combined during the study period. However, there were important variations by sex, age, cause and country. Specifically, unfavourable contributions of injury deaths were mainly observed in the USA, UK, and Canada. These findings highlight the need for targeted, country-specific injury prevention strategies to mitigate premature and unequal mortality.

**Supplementary Information:**

The online version contains supplementary material available at 10.1186/s12963-025-00409-6.

## Introduction

Most countries, particularly those with high incomes, have experienced a decline in their mortality rate due to significant development of socioeconomic indicators and healthcare services in the last two centuries, leading to higher life expectancy (LE) [1–4]. LE is often used as an important health metric to provide a summary of population health for a given country and to help policymakers develop policies based on LE trends. LE refers to the average number of years an individual is expected to live [5]. This measure facilitates the comparison of health profiles between countries [6, 7]. However, it does not reflect the variation in the length of life. Thus, to provide a comprehensive overview of a population’s health profile, an additional crucial health metric, life disparity (LD), which captures variation in age at the time of death, is used alongside LE. Utilizing both LE and LD is crucial to inform policymakers regarding life disparities across the age spectrum [5, 8].

The Group of Seven (G7) countries, consisting of Canada, France, Germany, Italy, Japan, the United Kingdom (UK), and the United States of America (USA), are among the most advanced economies of the world. With an average LE of 82 years, the G7 member states collectively accounted for nearly 30% of the global Gross Domestic Product (GDP) in 2023 [9]. The higher average LE in these countries is substantially attributable to higher economic growth, reforms in their national health systems, promotion of a healthy lifestyle [10, 11], higher GDP per capita, and lower infant mortality rate [12]. In the period between 2000 and 2020, among the G7 countries, Japan had the highest LE (84.7 years in 2020), and the USA experienced the lowest LE (77.4 years in 2020) compared to the rest of the G7 countries [13]. Despite the prominent growth of LE in these countries, the progress over the decade has not matched that of previous decades, and some inequalities have been observed in the LE of some countries, particularly in the USA and the UK [14, 15].

Injuries are among the important avoidable causes of death that substantially affect LE and LD of the population across both sexes and the age spectrum [16]. They significantly contribute to the mortality rate worldwide. In fact, recent studies have highlighted the impact of injury deaths on LE and LD in various contexts, considering different indicators including sex and age [17–20].

Despite the high level of economic development in the G7 countries, challenges caused by preventable injuries persist, and injury-related deaths remain a public health concern in all member states. These include a wide range of injuries, from traffic accidents to suicides and homicides [21]. Traffic accidents continue to be a major cause of injury-related deaths in G7 countries and remain a significant public health concern. However, other types of unintentional injuries, particularly poisonings and falls, have increased substantially and have now surpassed traffic accidents as the leading causes of unintentional injury deaths, especially in both the USA and Canada. For instance, drug poisoning, particularly those involving opioids and sedatives, has emerged as a dominant cause of unintentional injury deaths [22–24]. Additionally, Injuries impose a significant economic burden on societies. In Canada, for example, the preventable injuries caused an economic burden of $29.4 billion in a year [25].

Trends in injury mortality have varied across high-income countries, reflecting differences in prevention policies and public health strategies. For example, both Japan and Germany have consistently reported lower rates of injury-related deaths, a pattern linked to strong national safety regulations, widespread health education, and effective enforcement measures [26]. In contrast, although Canada has seen an overall decline in injury mortality over time, challenges remain in reducing specific causes, most notably unintentional poisoning, which has emerged as a leading contributor to premature mortality, particularly among younger populations [27].

The contribution of injury deaths to LE and LD in the G7 countries has not been investigated thoroughly. Most studies focused on a specific cause of injury, for example, suicide [28–31], traffic accidents [32, 33], or fall injuries [34, 35], and not all causes of injury together. In this paper, we aim to examine the contribution of injury deaths to changes in LE and LD across all G7 countries by sex, age group, and cause over two decades.

## Methods

### Data

The annual data for the underlying causes of death by age and sex were obtained from the World Health Organization (WHO) mortality database, using the reports that countries annually provide to the WHO based on medically certified deaths registered in their Civil Vital Registration System [36]. We utilized two timeframes to observe the changes in LE and LD over time. For each timeframe, a 3-year interval mortality data was employed to mitigate the impact of random year-to-year fluctuations. For the first period, we used the data from 2001–2003. Due to the unavailability of data, we used 2003–2005 as the first period for Italy. For the second period the data from 2017–2019 was used. We excluded the data from 2020 to avoid the impact of COVID-19 on our estimates. The extracted data has been coded based on the 10th revision of the International Classification of Diseases (ICD-10) and categorized by age group (< 1, 1–4, 5–9, 10–14,…, > 85), sex, and underlying cause of death. Injury-related deaths were categorized into three main classifications, with ten subcategories based on the WHO’s list of injury causes of death: unintentional (transport, poisoning, fall, fire, drowning, other), intentional (self-harm, assault, other), and undetermined [37]. While the WHO’s cause-of-death classification excludes X41, X42, X44, and X45 codes from the injury category and reclassifies them under “alcohol and drug use disorders” to minimize intent misclassification and enable focused surveillance of substance-related mortality, in this study, we defined unintentional poisonings using ICD-10 codes X40–X49. This approach aligns with standard practice in injury surveillance and public health reporting by agencies such as the CDC, PHAC, and Eurostat, and reflects the critical contribution of these causes to injury mortality trends, particularly in North America. The remaining causes of death were pooled together in a single group as “noninjury”. The details of specific causes and their associated ICD-10 codes can be found in Table A.1 in the Supplementary. The dataset used in this study are publicly available from the WHO Mortality Database (https://www.who.int/data/data-collection-tools/who-mortality-database) and all estimates are provided in the Supplementary.

### Statistical analysis

We calculated LE at birth using abridged life Table [38] for two time periods. To measure LD, we compute e-dagger ($\:{e}^{\dagger}$), representing the average remaining life expectancy at death, or the average years of life lost due to premature mortality [39]. LD was determined using Eq. 1:

1 $$\:{e}^{\dagger}={\int\:}_{0}^{m}e\left(x\right)d\left(x\right)dx,$$

where, $\:d\left(x\right)$ is the life table distribution of deaths $\:\left(\sum\:d\left(x\right)=1\right)$, $\:x$ denotes age, $\:e\left(x\right)$ indicates the LE at age $\:x$, and $\:m$ represents the maximum lifespan in the population. We assessed changes in LE and LD for each country, using the first period as the reference. Assuming that the effects of age and cause of death on changes in LE and LD are additive, we applied the continuous-change model to decompose these changes by age and cause [40]. This model assumes that age and cause of death vary continuously over time and proportionally. This approach allows us to estimate how variations in age or cause of death influence LE or LD changes over time. Using this method, changes in LE (LD) can be expressed as:

2 $$\:{y}_{s}-{y}_{f}=\sum\:_{i=1}^{n}{\int\:}_{{x}_{i\left(f\right)}}^{{x}_{i\left(s\right)}}\frac{\partial\:y}{\partial\:{x}_{i}}{dx}_{i}=\sum\:_{i=1}^{n}{c}_{i}$$

Here, $\:{y}_{s}\:$and $\:{y}_{f}$ denote the LE (LD) for the second and first period, respectively;$\:\:n$ represents the number of age groups; $\:{x}_{i}$ is cause-specific mortality for the $\:i$th age group, and $\:{c}_{i}$ indicates the contribution from each age and cause to changes in LE(LD). A reduction in cause-specific mortality (e.g., injury-related deaths) results in a positive contribution to LE (indicating increased longevity). For LD, a reduction in injury-related death below the threshold age (the age at which a death contributes neutrally to life disparity) results in a reduction in LD, and among those above the threshold age results in an increase in LD [41]. Data analysis was conducted in RStudio using open-source code from: https://github.com/jmaburto.

## Results

### LE and LD and their changes in G7 countries over the study period

Table 1 presents the changes in LE and LD for females and males in the G7 countries over two periods: 2001–2003 (2003–2005 for Italy) and 2017–2019. The findings revealed differing trends in LE and LD between females and males in these countries. In both periods, Japan consistently recorded the highest LE for both sexes, while the USA had the lowest. Over the study period, which spanned nearly two decades, the average LE increased by 2.12 years for females and 2.73 years for males in the G7 countries combined. In general, the changes in LE were more pronounced for males than for females. France experienced the highest increases in LE (2.87/4.18 years for females/males), while the USA experienced the lowest (1.77/2.01 years for females/males). Overall, LD widened for both sexes during the study period, with the USA experiencing the highest LD increase among males and France leading the increase for females. In contrast, Japan led in LD reduction for both sexes.

**Table 1:** Life expectancy (LE) and life disparity (LD) in G7 countries and their changes over time

		LE			LD		
		2001–2003*	2017–2019	Change	2001–2003*	2017–2019	Change
**Females**							
Canada		82.10	84.33	2.23	12.34	12.54	0.20
France		82.89	85.77	2.87	11.80	12.21	0.41
Germany		81.27	83.35	2.08	11.52	11.35	−0.17
Italy		83.25	85.27	2.03	11.27	11.24	−0.03
Japan		85.66	87.92	2.26	12.58	12.33	−0.25
UK		80.41	83.09	2.68	11.97	11.82	−0.16
USA		79.55	81.32	1.77	12.92	13.09	0.17
**G7 combined**		81.60	83.72	2.12	12.32	12.57	0.25
**Males**							
Canada		76.98	80.04	3.06	12.73	13.12	0.38
France		75.54	79.72	4.18	13.44	13.09	−0.35
Germany		75.31	78.65	3.34	12.66	12.40	−0.26
Italy		77.51	80.78	3.26	12.27	11.86	−0.41
Japan		78.21	81.38	3.17	12.74	12.20	−0.54
UK		75.73	79.42	3.69	12.58	12.54	−0.03
USA		74.30	76.31	2.01	13.93	14.46	0.53
**G7 combined**		75.72	78.46	2.73	13.27	13.43	0.16

*The first period was 2001–2003 for all countries except Italy, where it was 2003–2005

### Overall contributions of injury deaths to changes in LE and LD

The contributions of injury deaths to changes in LE and LD between the two study periods are presented in Table 2. The results suggest that injury deaths contributed more to LE increases and LD reductions for males than for females in the G7 countries. Overall, no significant contribution of injury deaths to changes in LE or LD was observed for either sex across the G7 countries combined. Specifically, injury deaths contributed to a 0.03-year decrease in LE and a 0.02-year increase in LD for females, while no change in LE and a 0.01-year reduction in LD were observed for males.

**Table 2:** Contribution of injury deaths to changes in life expectancy (LE) and life disparity (LD) in G7 countries

		LE		LD	
		Total change	Injury death contribution	Total change	Injury death contribution
**Females**					
Canada		2.23	−0.10	0.20	0.01
France		2.87	0.38	0.41	−0.11
Germany		2.08	0.10	−0.17	−0.16
Italy		2.03	0.13	−0.03	−0.04
Japan		2.26	0.24	−0.25	−0.10
UK		2.68	−0.03	−0.16	0.00
USA		1.77	−0.23	0.17	0.11
**G7 combined**		2.12	−0.03	0.25	0.02
**Males**					
Canada		3.06	−0.09	0.38	0.03
France		4.18	0.64	−0.35	−0.37
Germany		3.34	0.37	−0.26	−0.33
Italy		3.26	0.38	−0.41	−0.26
Japan		3.17	0.58	−0.54	−0.33
UK		3.69	0.02	−0.03	−0.07
USA		2.01	−0.42	0.53	0.22
**G7 combined**		2.73	−0.00	0.16	−0.01

Note: The first period was 2001-03, except for Italy, where it was 2003-05, and the second period was 2017-19

Among females, France experienced the largest increases in LE attributable to injury deaths in both absolute terms (0.38 years) and relative terms, with injury deaths contributing 13% to the total LE increase. In contrast, injury deaths contributed 0.23 years (13%) to the LE decrease in the USA. For males, Japan showed the largest rise in LE, with 18% of the total LE increase attributable to the injury deaths. Whereas the USA experienced the highest negative contributions from injuries to LE among the G7 countries, with a 0.42-year reduction. Regarding LD, injury deaths contributed to LD reductions in all G7 countries except Canada and the USA, where slight increases in LD were observed (0.03 and 0.22 years, respectively).

### Age-specific contributions of injury deaths to changes in LE and LD

**Fig. 1:**
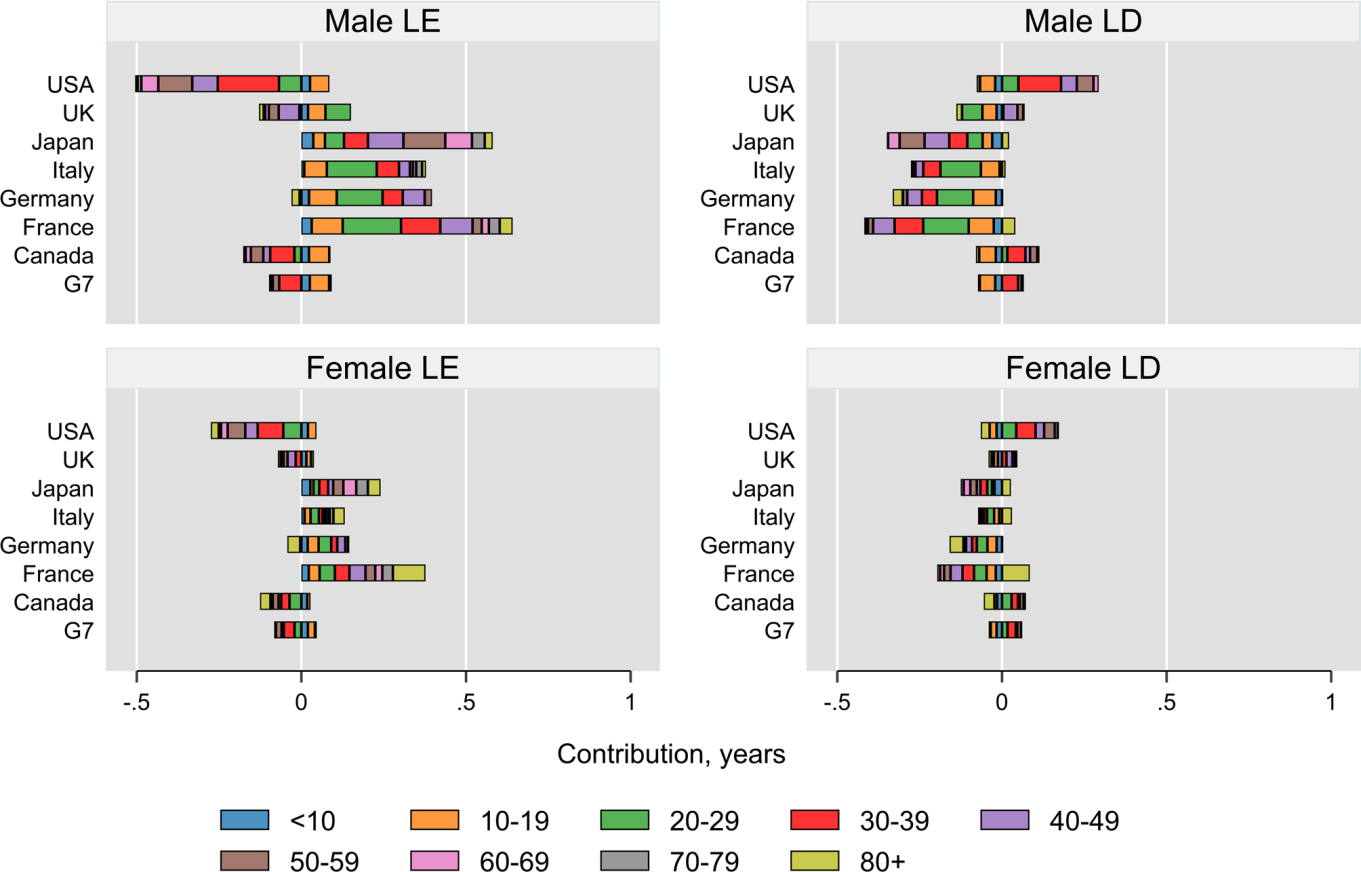
Age-specific contributions of injury deaths to the changes in life expectancy (LE) and life disparity (LD) across the G7 countries

Figure 1 shows age-specific contributions of injury deaths to changes in LE and LD in the G7 countries. Among females, injury deaths consistently contributed to LE improvement across all age groups in France, Italy and Japan. The highest absolute positive contribution to changes in LE was observed in France in the 80 + age group (0.1 year). In contrast, the highest absolute negative contribution was observed in the USA among those aged 30–39 (–0.08 years).

**Fig. 2:**
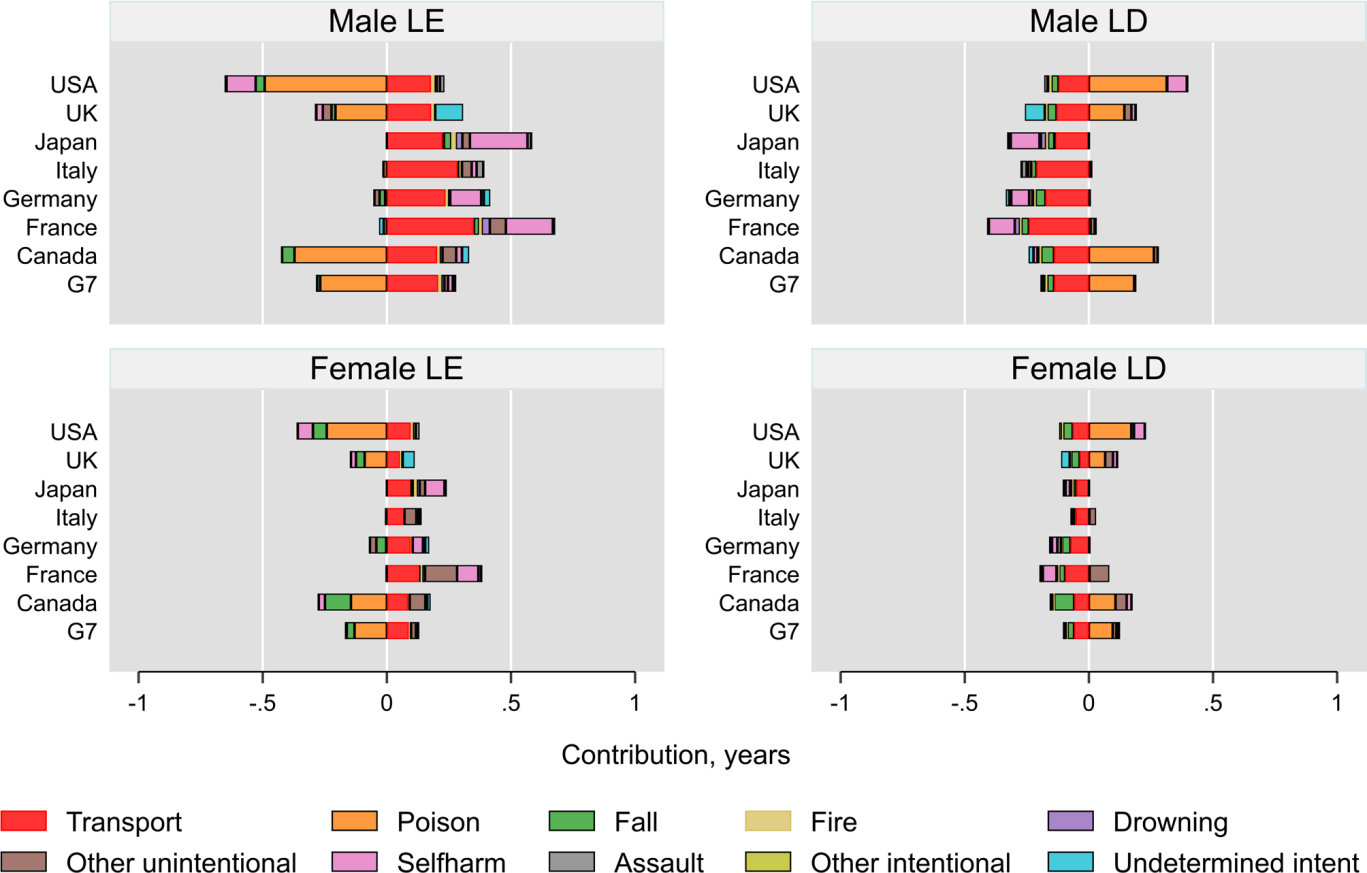
Cause specific contributions of injury deaths to changes in life expectancy in G7 countries

Similar patterns were observed among males, where injury deaths contributed to improvements in LE across all age groups in France, Italy, and Japan. The largest absolute increase was seen in the 20–29 age group in France (0.18 years), while the greatest negative contribution occurred in the 30–39 age group in the USA (–0.19 years). (Fig. 2)

Among females, the results for LD changes showed improvements across all age groups in Japan, France, and Italy, except in the 80 + age group, where the highest contribution to LD was also observed in this age group. A striking absolute increase in LD, indicating a worsening trend, was observed among females in the 30–39 age group in the USA. Among males, the absolute contributions to LD were more pronounced. Notably, Germany was the only country where no age groups experienced a worsening in LD due to changes in injury deaths. The 20–29 age group in France experienced the greatest improvement in LD, while the 30–39 age group in the USA showed the most pronounced worsening in LD.

### Cause-specific contributions of injury deaths to changes in LE and LD

Regarding the cause-specific contributions, a consistent pattern was observed across countries. Transport accidents were the leading contributors to improvements in LE among females in all countries. This was followed by gains associated with reductions in self-harm in Italy, Germany, France, and Japan. In these four countries, no specific cause notably contributed to a decrease in LE. However, unintentional poisoning stood out as the primary contributor to declining LE in the USA, UK, and Canada. This was followed by self-harm in the UK and USA, and by falls in Canada. A similar pattern was observed among males, with generally more pronounced contributions. However, falls contributed to reductions in LE more significantly among females than males in Canada, the USA, the UK, and Germany, with Canada showing the highest contributions.

Except for females in Canada, transport accidents had the greatest contributions to reductions in LD for both sexes across all countries. Improvements in LD due to self-harm were observed in Japan, France, Italy, and Germany for both sexes. In contrast, unintentional poisoning in the USA, the UK and Canada, was associated with worsening LD.

### Age- and cause-specific contributions of injury deaths to LE and LD

**Fig. 3:**
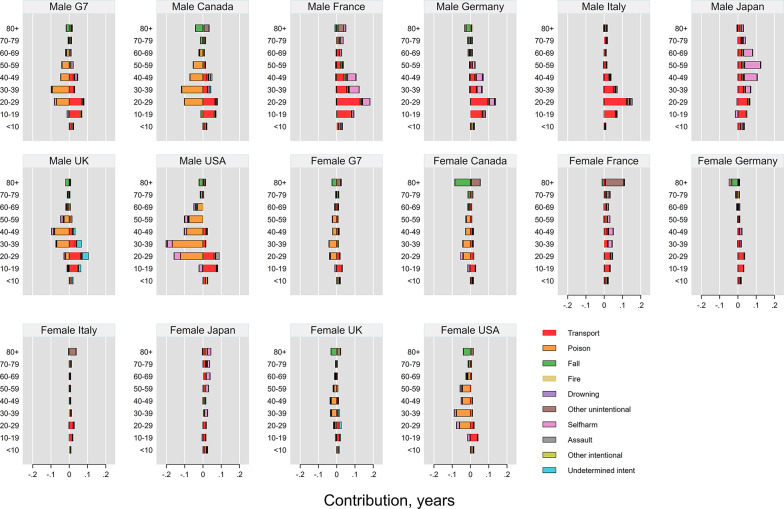
Age- and cause-specific contributions of injury deaths to changes in life expectancy across the G7 countries

For both sexes, transport accidents had the highest positive impact on LE, particularly among individuals aged 15–24, spanning the 10–19 and 20–29 age groups (Fig. 3). In contrast, unintentional poisoning had a more negative impact on LE among individuals aged 25–35 in the USA and Canada, and among those aged 35–45 in the UK, spanning the 20–29 and 30–39 age groups in the former, and 30–39 and 40–49 in the latter.

**Fig. 4:**
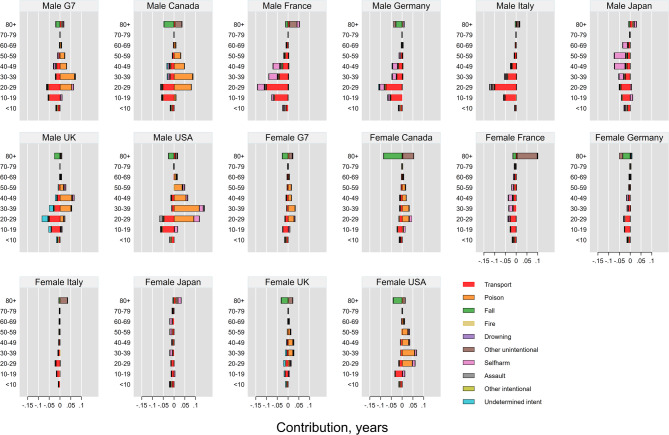
Age- and cause-specific contributions of injury deaths to changes in life disparity across the G7 countries

Reductions in LD due to falls were observed in all countries among individuals aged 85 and above (Fig. 4; detailed results are provided in Tables A2–A33 in the Supplementary File). LD reductions due to transport-related deaths were evident in the 10–29 age group, particularly in Germany and France. In Germany, additional LD gains were observed among those aged 85 + years, primarily due to declines in falls and other unintentional injuries. In contrast, the USA experienced a worsening of LD among individuals aged 20–39, largely driven by unintentional poisoning. A similar pattern of LD worsening was seen in Italy and France among those aged 85+, associated with other unintentional injuries. Meanwhile, in France, improvements in LD were also linked to reductions in self-harm among individuals aged 30–49.

## Discussion

This study presents a comprehensive analysis of the contributions of injury deaths to the changes in LE and LD among the G7 countries since the turn of the new millennium. A cross-country analysis was employed by examining the contributions of age and injury causes of deaths stratified by sex. The findings indicate that, overall, injury deaths had negligible contributions to changes in LE or LD when considering G7 countries combined. This was mainly driven by negative contributions of unintentional poisoning which offset positive contributions of other injury-related causes especially transport accidents. Moreover, country-specific patterns reveal notable variations. France and the USA showed the most significant contributions, positive and negative, respectively, for both sexes. In these countries, injury deaths had a more visible impact on both improving and worsening LE and LD across certain age groups.

Historically, the baseline mortality rate from injuries has been higher in males than in females, largely due to a greater tendency for risk-taking behaviours among males [42]. This may help explain why reductions in injury deaths have a larger impact on increasing LE in males compared to females. Research in the USA has shown that LE increases among males are approximately twice that of females due to a reduction in injury deaths [43].

We found that in all G7 countries, transport accidents had the largest positive contributions to LE over time, particularly among younger males. This could be a result of the improvement in the implementation of road traffic strategy plans in these countries. For example, in Canada, despite a growing road user population, the implementation of road safety plans led to a reduction in fatalities and serious injuries from road traffic accidents between 2001 and 2019 [44, 45]. Based on the results of this study, the reduction was even more notable in other G7 countries, particularly European counterparts such as France and Germany. For instance, France recorded a significant 44% reduction in mortality rates from road traffic accidents between 2002 and 2008. This resulted from the implementation of increased monitoring and enforcement measures, such as utilizing automatic speed radars [32]. These regulations, specifically those addressing speeding and alcohol consumption, are more beneficial for younger male adults, who are more prone to engaging in risky behaviours. For instance, using speed cameras as traffic control enforcement has led to a reduction of 35% in fatalities and injuries at camera sites in the UK [46]. These strategies could significantly reduce accident rates, ultimately leading to increased LE and reduced LD.

Our findings regarding changes in LD offer insights into the inequality in the distribution of age at death. LD captures the overall variability in age at death across the population and is especially sensitive to premature mortality. What we observed is that injury deaths contributed to a reduction in inequality at the age of death due to more favourable changes in injury deaths among younger individuals than older ones (e.g., larger reductions in younger individuals than older ones). Because deaths at younger ages have a greater impact on LD, improvements in younger-age injury mortality, such as declines in self-harm or transport-related fatalities, lead to reductions in lifespan variation. This is consistent with findings by Borboa et al., who emphasize the role of early-age mortality in shaping lifespan inequality [47]. These results underscore the value of injury prevention strategies that target premature deaths in younger populations, promoting a more equitable distribution of longevity [48].

Falls have increased considerably due to the growing older adult population, particularly among those with frailty [49]. Although falls are among the preventable causes of death and fall prevention programs have made progress globally, the increase in fall incidents remains a challenge that all countries are facing to some extent [34, 49]. Our findings revealed that, among the G7 countries, Canadian females and males experienced the largest drop in LE due to fall injury deaths. In Canada, 20–30% of older individuals experience at least one fall annually [35]. Between 2003 and 2010, the mortality rate due to falls increased by 65%, making it the leading cause of injury deaths among older Canadians [35]. This trend continued from 2010 to 2022 with a sharp increase from 2017 to 2022 [50]. To prevent falls among Canadian older people, the Government of Canada has implemented guidelines or prevention plans, such as providing organizations and health care teams with fall prevention strategies and e-learning programs specifically designed to improve the knowledge and skills required for fall prevention in older adults’ care centres and home care [35]. Nonetheless, Canada still ranks last among the G7 countries in reducing falls’ negative contributions to LE, highlighting the need for further action.

Our results also revealed variations in the contributions of injury deaths across countries. For instance, the UK and the USA were the only countries where self-harm deaths contributed to the LE decreases in both sexes. On the contrary, Japan witnessed a large gain in LE due to self-harm fatalities, and it was the only G7 country where self-harm had a greater contribution to the LE increases than transport accidents among males. The rate of self-harm is increasing in the USA and Canada, particularly among adolescents and young adults, who are in their critical period of transition from adolescence to adulthood, resulting in suicide being the leading cause of death among youths aged 15–24 years old [51, 52]. Notably, the USA is experiencing a greater impact of self-harm on LE, particularly among youths, as suicide rates among young people have risen significantly since 2010 [31, 53]. In contrast, self-harm statistics in France present a somewhat different picture, with research highlighting lower rates compared to Canada, the UK and the USA, possibly due to implementing different preventive strategies such as region-specific prevention programs and varying levels of mental health support [30]. The lower rates of self-harm in countries like France, Germany, and Japan may reflect different societal norms, access to healthcare, attitude toward mental health, or national preventive measures [28]. For instance, insurance coverage and the cost of healthcare in the USA are considered a barrier to access to mental health services [54–56]. Conversely, the healthcare system in European countries, such as Germany, offers better continuity of mental healthcare, which facilitates access to care [57]. Another possible factor contributing to this trend is substance use, which has been proven to be associated with increased rates of self-harm through various psychological and behavioural mechanisms [58]. Research shows that substance use, particularly of cannabis and cocaine, is considerably higher in the USA than in other countries, which may partly explain the higher rates of self-harm observed in the USA [59]. In this study, unintentional poisonings, particularly those involving drug and alcohol use, emerged as major contributors to declining LE and worsening LD in the USA, Canada, and the UK, with the most pronounced effects observed in the USA. The growing burden of substance-related mortality, including opioid overdoses and alcohol-related poisonings, has been identified as a key driver of premature and unequal mortality patterns [60]. These deaths are disproportionately concentrated among younger and middle-aged adults, whose premature loss not only lowers average LE but also increases variability in age at death, thereby widening LD [61]. In the USA in particular, the opioid epidemic has reversed decades of progress in mortality reduction among working-age populations, contributing to a phenomenon described as “deaths of despair” [61]. Similar trends have been documented in Canada and the UK [62, 63], where toxic drug supplies and alcohol misuse have contributed to rising injury mortality in recent years. These findings underscore the importance of addressing substance use and related social determinants through targeted prevention, harm reduction, and mental health services in order to improve population health and reduce mortality inequality.

The findings of this study highlight several policy implications regarding various causes of injury deaths, including but not limited to unintentional positioning, transport accidents, falls and self-harm. Implementing targeted strategies for transport accidents and self-harm, particularly in younger males, as well as addressing falls among older adults, could largely contribute to LE increase and LD reduction in the G7 countries. A study conducted in Nordic countries showed that despite the existing national suicide prevention programs, tailored strategies for younger adults remain necessary. Additionally, the study suggested that embedding exercise-based initiatives into routine preventative health care for older adults may be effective in reducing fall-related deaths in this population [20].

A cross-country analysis reveals important disparities in how injury deaths shape LD across G7 countries. While Germany and France experienced consistent reductions in LD due to declines in injury deaths, particularly among younger adults, other countries, notably the USA, Canada, and the UK, saw an increase in LD linked to rising mortality from unintentional poisonings. These deaths, often concentrated among adults aged 25–44, disproportionately drive premature mortality and, consequently, greater variation in age at death. In the USA, for example, injury deaths contributed to a 0.22-year increase in LD for males, the highest among the G7, primarily reflecting the burden of drug- and alcohol-related poisonings. In contrast, countries such as Japan and Germany showed more balanced reductions across age groups, leading to greater LD compression. The observed heterogeneity suggests that national policy responses, such as substance use prevention, mental health services, and injury surveillance systems, play a critical role in mediating the effects of injury deaths on lifespan inequality.

We acknowledge that our study has some limitations. Cross-country comparisons could be affected by the potential variability in the reporting practices and data quality. The WHO mortality database relies on the medically certified death reports from each member country, which could be subject to diagnostic inaccuracies, coding errors and underreporting. For instance, a notable limitation of our analysis relates to the potential misclassification of unintentional injury deaths, particularly among older adults. Within the unintentional injury group, deaths due to “exposure to unspecified factors” (ICD-10 code: X59) were the leading cause of death, which lacks detail on the exact mechanism of injury. Previous research in Norway [64] has shown that most X59-coded deaths were likely misclassified fall-related fatalities.

Additionally, suffocation deaths, another subcategory of unintentional injuries, may include misclassified suicides, particularly among the elderly, where diagnostic ambiguity is more common. Compounding this issue, prior research has shown that mortality patterns for falls among older adults can resemble those of influenza-associated deaths. During seasonal influenza peaks, increased mortality in this age group may not always be attributed to respiratory causes and could be recorded as unintentional injuries, including falls [65]. These issues suggest that the mortality are underestimated for some injury causes (e.g. suicide) while overestimated for other injury causes (e.g. unintentional injuries). Specifically, if there are temporal trends in these misclassifications over time, then our estimates on the contributions of injury-specific causes to changes in LE/LD would be biased.

Differences in healthcare systems as well as socioeconomic and cultural factors in each country may not be fully reflected in our analysis. Furthermore, this study included a comparative analysis of age- and cause-specific injury deaths in measuring LE and LD over two distinct time periods in G7 countries. While the study offers a valuable summary of these indicators and their impact on changes across these countries, the complexity and interrelation of these indicators may not have been fully captured in cross-country analysis. Finally, we used the term “sex” as it appears in death registration records, which typically refer to legal sex as recorded at the time of death. We recognize that definitions and data availability may vary by jurisdiction.

## Conclusion

Injury deaths made negligible overall contributions to changes in LE and LD across the G7 countries combined during the study period. However, there were substantial variations by cause, age and sex as well as across countries. While transport accidents contributed positively, unintentional poisonings, particularly in the USA, UK, and Canada, had substantial negative effects, especially among younger age groups. These findings highlight the need for targeted, country-specific injury prevention strategies to mitigate premature and unequal mortality.

## Supplementary Information

Supplementary Material 1

## Data Availability

“Data used in the study is publicly available at WHO website.”

## Abbreviations

G7: The Group of Seven
LD: Life disparity
LE: Life expectancy
UK: United Kingdom
USA: United States of America
WHO: World Health Organization
