# Supplementary material for: Contributions of injury deaths to changes in life expectancy and disparity: A comparative analysis of G7 countries over two decades
Source: Popul Health Metr. 2025 Aug 4;23:43. doi: 10.1186/s12963-025-00409-6 (PMC12323151; doi:10.1186/s12963-025-00409-6)
Supplement: Supplementary file 1 — Supplementary Material 1 [file 12963_2025_409_MOESM1_ESM.pdf]

Online Supplementary File

Table A.1: Specific causes of deaths and their associated ICD-10 codes

Cause	ICD-10 codes
1. Unintentional injuries	
1.1 Transport accidents	V01-V99
1.2 Poisonings	X40-X49
1.3 Falls	W00-W19
1.4 Fires	X00-X09
1.5 Drownings	W65-W74
1.6 Other unintentional injuries	W20-W64, W75-W99, X10-X39, X50-X59, Y40-Y86, Y88-Y89
2. *Intentional injuries*	
2.1 Self-harm	X60-X84
2.2 Assault	X85-Y09
2.3 Other intentional injuries	Y35-Y36, Y87
3. *Injuries with undetermined intent*	Y10-Y34

Table A.2: Contributions of injury deaths to changes in LE for females in Canada

Age groups	Age contribution	Transport	Poison	Fall	Fire	Drowning	Other unintentional	Self-harm	Assault	Other intentional	Undetermined intent	Non injury	Total Injury
<1	0.051	0.001	0.000	0.000	0.000	0.000	0.001	0.000	0.001	0.000	0.000	0.048	0.003
1-4	0.019	0.003	0.000	0.000	0.001	0.001	0.002	0.000	0.002	0.000	0.000	0.011	0.008
5-9	0.007	0.005	0.000	0.000	0.001	0.000	0.001	0.000	0.000	0.000	0.000	0.001	0.006
10-14	0.009	0.007	-0.001	0.000	0.000	0.001	0.000	-0.004	0.000	0.000	0.000	0.006	0.003
15-19	0.016	0.017	-0.007	0.000	0.000	0.001	0.001	-0.007	0.001	0.000	0.000	0.009	0.007
20-24	-0.007	0.013	-0.017	0.000	0.000	0.000	0.000	-0.008	0.001	0.000	0.001	0.004	-0.011
25-29	-0.026	0.003	-0.023	0.000	0.001	-0.001	-0.001	-0.007	0.000	0.000	0.002	-0.001	-0.025
30-34	-0.016	0.004	-0.023	0.000	0.001	0.000	0.000	0.001	-0.001	0.000	0.002	0.000	-0.017
35-39	0.010	0.005	-0.017	-0.001	0.000	0.000	0.001	0.002	0.000	0.000	0.002	0.019	-0.009
40-44	0.040	0.005	-0.013	0.001	0.000	0.001	0.000	0.001	0.001	0.000	0.002	0.043	-0.003
45-49	0.069	0.005	-0.013	-0.001	0.000	0.000	0.000	0.002	0.000	0.000	0.002	0.074	-0.005
50-54	0.087	0.003	-0.014	-0.001	0.000	0.000	-0.001	0.000	0.000	0.000	0.001	0.098	-0.011
55-59	0.112	0.003	-0.009	-0.001	0.000	0.000	0.000	-0.001	0.000	0.000	0.001	0.119	-0.007
60-64	0.169	0.003	-0.004	-0.002	0.000	0.000	0.000	-0.002	0.000	0.000	0.001	0.173	-0.005
65-69	0.225	0.003	-0.002	-0.004	0.000	0.000	0.001	-0.001	0.000	0.000	0.001	0.226	-0.002
70-74	0.243	0.003	-0.001	-0.005	0.000	0.000	0.002	0.000	0.000	0.000	0.000	0.244	-0.001
75-79	0.307	0.002	0.000	-0.007	0.000	0.000	0.006	0.000	0.000	0.000	0.000	0.306	0.000
80-84	0.377	0.002	0.000	-0.014	0.000	0.000	0.007	0.000	0.000	0.000	0.000	0.382	-0.005
≥85	0.537	0.001	0.000	-0.071	0.000	0.000	0.042	0.000	0.000	0.000	0.000	0.564	-0.027
Total	**2.228**	**0.086**	**-0.145**	**-0.105**	**0.006**	**0.000**	**0.063**	**-0.025**	**0.008**	**0.000**	**0.014**	**2.326**	**-0.098**

Table A.3: Contributions of injury deaths to changes in LE for females in France

Age groups	Age contribution	Transport	Poison	Fall	Fire	Drowning	Other unintentional	Self-harm	Assault	Other intentional	Undetermined intent	Non injury	Total Injury
<1	0.035	0.001	0.000	0.000	0.000	0.000	0.001	0.000	0.000	0.000	0.000	0.031	0.004
1-4	0.029	0.004	0.000	0.001	0.001	0.002	0.001	0.000	0.002	0.000	0.000	0.017	0.013
5-9	0.015	0.004	0.000	0.000	0.000	0.001	0.001	0.000	0.001	0.000	0.000	0.010	0.006
10-14	0.017	0.007	0.000	0.000	0.000	0.000	0.000	0.000	0.000	0.000	0.000	0.010	0.008
15-19	0.039	0.023	0.000	0.000	0.000	0.000	0.000	0.000	0.001	0.000	0.000	0.014	0.025
20-24	0.042	0.020	0.000	0.000	0.000	0.000	0.001	0.004	0.001	0.000	0.000	0.014	0.027
25-29	0.034	0.012	-0.001	0.000	0.000	0.000	0.001	0.004	0.001	0.000	0.001	0.015	0.019
30-34	0.043	0.010	0.001	0.000	0.000	0.000	0.000	0.009	0.001	0.000	0.000	0.021	0.022
35-39	0.069	0.008	0.000	0.001	0.001	0.001	0.000	0.012	0.001	0.000	0.000	0.047	0.022
40-44	0.105	0.008	-0.001	0.001	0.001	0.001	0.002	0.014	0.001	0.000	0.000	0.077	0.027
45-49	0.114	0.006	-0.001	0.002	0.001	0.001	0.002	0.010	0.000	0.000	0.000	0.093	0.021
50-54	0.080	0.005	-0.001	0.001	0.000	0.001	0.001	0.007	0.001	0.000	-0.001	0.065	0.016
55-59	0.072	0.005	-0.001	0.001	0.000	0.001	0.001	0.006	0.000	0.000	0.000	0.058	0.014
60-64	0.035	0.005	-0.001	0.001	0.000	0.001	0.001	0.005	0.000	0.000	-0.001	0.024	0.011
65-69	0.111	0.005	0.000	0.001	0.000	0.000	0.001	0.004	0.000	0.000	-0.001	0.101	0.010
70-74	0.216	0.005	0.000	0.001	0.000	0.000	0.004	0.003	0.000	0.000	0.000	0.202	0.014
75-79	0.368	0.003	0.001	0.001	0.000	0.000	0.011	0.002	0.000	0.000	0.000	0.349	0.019
80-84	0.518	0.002	0.001	0.003	0.000	0.000	0.021	0.002	0.000	0.000	0.000	0.488	0.030
≥85	0.931	0.001	0.001	-0.014	0.000	0.000	0.079	0.002	0.000	0.000	0.000	0.862	0.069
Total	**2.873**	**0.135**	**-0.001**	**0.001**	**0.007**	**0.011**	**0.129**	**0.086**	**0.011**	**0.000**	**-0.002**	**2.496**	**0.377**

Table A.4: Contributions of injury deaths to changes in LE for females in Germany

Age groups	Age contribution	Transport	Poison	Fall	Fire	Drowning	Other unintentional	Self-harm	Assault	Other intentional	Undetermined intent	Non injury	Total Injury
<1	0.065	0.001	0.000	0.000	0.000	0.000	0.001	0.000	0.002	0.000	0.001	0.060	0.005
1-4	0.025	0.003	0.000	0.001	0.001	0.001	0.002	0.000	0.001	0.000	0.000	0.016	0.009
5-9	0.011	0.003	0.000	0.000	0.001	0.001	0.001	0.000	0.000	0.000	0.000	0.005	0.005
10-14	0.010	0.005	0.000	0.000	0.000	0.000	0.001	-0.001	0.000	0.000	0.000	0.003	0.006
15-19	0.034	0.024	0.000	0.000	0.000	0.000	0.000	0.001	0.000	0.000	0.001	0.008	0.027
20-24	0.033	0.018	0.001	0.000	0.000	0.000	0.000	0.002	0.001	0.000	0.001	0.010	0.024
25-29	0.022	0.009	0.000	0.000	0.000	0.000	0.000	0.004	0.001	0.000	0.001	0.007	0.014
30-34	0.010	0.004	-0.001	0.000	0.000	0.000	0.000	0.001	0.001	0.000	0.001	0.004	0.005
35-39	0.037	0.006	-0.001	0.000	0.000	0.000	0.000	0.005	0.001	0.000	0.001	0.023	0.013
40-44	0.083	0.004	0.000	0.000	0.000	0.000	0.000	0.005	0.001	0.000	0.001	0.070	0.013
45-49	0.118	0.004	0.000	0.001	0.000	0.000	0.000	0.005	0.001	0.000	0.001	0.107	0.011
50-54	0.124	0.003	-0.001	0.000	0.000	0.000	-0.001	0.001	0.001	0.000	0.001	0.119	0.004
55-59	0.068	0.002	0.000	-0.001	0.000	0.000	-0.002	0.003	0.000	0.000	0.001	0.064	0.004
60-64	0.015	0.002	0.000	-0.001	0.000	0.000	-0.002	0.003	0.000	0.000	0.000	0.013	0.002
65-69	0.103	0.002	0.000	-0.001	0.000	0.000	-0.002	0.003	0.000	0.000	0.000	0.101	0.002
70-74	0.216	0.002	0.000	-0.003	0.000	0.000	-0.003	0.002	0.000	0.000	0.000	0.218	-0.002
75-79	0.396	0.002	0.000	-0.003	0.000	0.000	-0.003	0.002	0.000	0.000	0.001	0.397	-0.002
80-84	0.412	0.001	0.000	-0.006	0.000	0.000	-0.004	0.002	0.000	0.000	0.001	0.416	-0.005
≥85	0.297	0.000	0.000	-0.027	0.000	0.000	-0.013	0.002	0.000	0.000	0.003	0.332	-0.034
Total	**2.078**	**0.096**	**-0.003**	**-0.039**	**0.005**	**0.002**	**-0.026**	**0.041**	**0.009**	**0.000**	**0.017**	**1.975**	**0.103**

Table A.5: Contributions of injury deaths to changes in LE for females in Italy

Age groups	Age contribution	Transport	Poison	Fall	Fire	Drowning	Other unintentional	Self-harm	Assault	Other intentional	Undetermined intent	Non injury	Total Injury
<1	0.089	0.001	0.000	0.000	0.000	0.000	0.001	0.000	0.000	0.000	0.000	0.087	0.001
1-4	0.017	0.002	0.000	0.000	0.000	0.001	0.001	0.000	0.000	0.000	0.000	0.012	0.005
5-9	0.011	0.004	0.000	0.000	0.000	0.000	0.000	0.000	0.000	0.000	0.000	0.007	0.004
10-14	0.006	0.003	0.000	0.000	0.000	0.000	0.000	-0.001	0.000	0.000	0.000	0.004	0.002
15-19	0.023	0.014	0.000	0.001	0.000	0.000	0.001	-0.001	0.001	0.000	0.000	0.007	0.016
20-24	0.023	0.013	0.000	0.000	0.000	-0.001	0.002	0.001	0.001	0.000	0.000	0.007	0.016
25-29	0.021	0.007	-0.001	0.000	0.000	0.000	0.001	0.001	0.001	0.000	0.000	0.011	0.010
30-34	0.023	0.005	0.000	0.000	0.000	0.000	0.000	0.001	0.000	0.000	0.000	0.016	0.006
35-39	0.030	0.003	0.000	0.000	0.000	0.000	0.000	0.001	0.001	0.000	0.000	0.024	0.005
40-44	0.052	0.002	0.000	0.000	0.000	0.000	0.001	0.000	0.000	0.000	0.000	0.047	0.004
45-49	0.062	0.003	0.000	0.000	0.000	0.000	0.000	0.000	0.001	0.000	0.000	0.058	0.004
50-54	0.068	0.001	0.000	0.001	0.000	0.000	0.001	0.001	0.000	0.000	0.000	0.064	0.003
55-59	0.083	0.002	0.000	0.000	0.000	0.000	-0.001	0.001	0.000	0.000	0.000	0.079	0.004
60-64	0.077	0.002	0.000	0.000	0.000	0.000	0.000	0.000	0.000	0.000	0.000	0.075	0.002
65-69	0.141	0.003	0.000	0.000	0.000	0.000	0.000	0.002	0.000	0.000	0.000	0.136	0.005
70-74	0.189	0.002	0.000	0.000	0.000	0.000	0.001	0.001	0.000	0.000	0.000	0.185	0.004
75-79	0.308	0.002	0.000	0.000	0.000	0.000	0.005	0.001	0.000	0.000	0.000	0.300	0.008
80-84	0.340	0.001	0.000	0.000	0.000	0.000	0.008	0.001	0.000	0.000	0.000	0.330	0.010
≥85	0.468	0.000	0.000	-0.004	0.000	0.000	0.028	0.001	0.000	0.000	0.000	0.444	0.025
Total	**2.029**	**0.069**	**-0.003**	**-0.002**	**0.001**	**0.001**	**0.048**	**0.010**	**0.007**	**0.000**	**0.002**	**1.897**	**0.132**

Table A.6: Contributions of injury deaths to changes in LE for females in Japan

Age groups	Age contribution	Transport	Poison	Fall	Fire	Drowning	Other unintentional	Self-harm	Assault	Other intentional	Undetermined intent	Non injury	Total Injury
<1	0.093	0.001	0.000	0.000	0.000	0.000	0.003	0.000	0.001	0.000	0.000	0.087	0.005
1-4	0.026	0.004	0.000	0.001	0.001	0.003	0.001	0.000	0.001	0.000	0.000	0.015	0.012
5-9	0.017	0.006	0.000	0.000	0.001	0.001	0.000	0.000	0.001	0.000	0.000	0.006	0.010
10-14	0.006	0.002	0.000	0.000	0.001	0.000	0.000	-0.003	0.001	0.000	0.000	0.006	0.000
15-19	0.018	0.011	0.000	0.000	0.001	0.000	0.000	-0.004	0.000	0.000	0.000	0.010	0.009
20-24	0.018	0.010	0.000	0.000	0.001	0.001	0.000	-0.003	0.000	0.000	0.000	0.010	0.009
25-29	0.019	0.004	0.000	0.001	0.000	0.000	0.000	0.003	0.001	0.000	0.000	0.010	0.009
30-34	0.032	0.003	0.000	0.001	0.000	0.000	0.000	0.008	0.001	0.000	0.001	0.018	0.014
35-39	0.041	0.003	0.000	0.001	0.000	0.000	0.000	0.007	0.000	0.000	0.001	0.028	0.013
40-44	0.045	0.003	0.000	0.000	0.000	0.001	0.000	0.003	0.000	0.000	0.000	0.036	0.009
45-49	0.049	0.003	0.000	0.000	0.001	0.001	0.000	0.002	0.000	0.000	0.000	0.042	0.007
50-54	0.084	0.004	0.000	0.001	0.001	0.001	0.000	0.005	0.000	0.000	0.000	0.072	0.012
55-59	0.102	0.006	0.000	0.001	0.000	0.001	0.000	0.010	0.000	0.000	0.000	0.083	0.019
60-64	0.127	0.006	0.000	0.001	0.000	0.001	0.000	0.011	0.001	0.000	0.000	0.108	0.019
65-69	0.185	0.007	0.000	0.001	0.000	0.001	0.001	0.010	0.000	0.000	0.000	0.165	0.020
70-74	0.258	0.008	0.000	0.002	0.000	0.001	0.001	0.006	0.000	0.000	0.000	0.240	0.018
75-79	0.360	0.007	0.000	0.002	0.001	0.000	0.003	0.006	0.000	0.000	-0.001	0.343	0.017
80-84	0.437	0.005	0.000	0.002	0.001	-0.001	0.004	0.006	0.000	0.000	-0.001	0.420	0.017
≥85	0.340	0.006	0.000	-0.005	0.001	0.000	0.008	0.012	0.000	0.000	-0.001	0.318	0.022
Total	**2.259**	**0.099**	**0.00o**	**0.009**	**0.012**	**0.011**	**0.022**	**0.078**	**0.010**	**0.000**	**-0.001**	**2.018**	**0.241**

Table A.7: Contributions of injury deaths to changes in LE for females in the UK

Age groups	Age contribution	Transport	Poison	Fall	Fire	Drowning	Other unintentional	Self-harm	Assault	Other intentional	Undetermined intent	Non injury	Total Injury
<1	0.117	0.000	0.000	0.000	0.000	0.001	0.000	0.000	0.000	0.000	0.003	0.113	0.005
1-4	0.030	0.002	0.000	0.000	0.002	0.001	0.000	0.000	0.000	0.000	0.002	0.023	0.007
5-9	0.014	0.003	0.000	0.000	0.001	0.000	-0.001	0.000	0.000	0.000	0.001	0.010	0.004
10-14	0.015	0.004	0.000	0.000	0.000	0.000	0.000	-0.001	0.000	0.000	0.002	0.009	0.006
15-19	0.026	0.011	-0.001	0.000	0.000	0.000	-0.001	-0.006	0.000	0.000	0.005	0.017	0.008
20-24	0.029	0.008	-0.002	0.000	0.001	0.000	-0.001	-0.002	0.000	0.000	0.006	0.019	0.009
25-29	0.020	0.005	-0.007	0.000	0.000	0.000	-0.002	-0.002	0.000	0.000	0.006	0.021	0.000
30-34	0.016	0.003	-0.011	0.000	0.000	0.000	-0.002	-0.002	0.000	0.000	0.004	0.022	-0.006
35-39	0.010	0.002	-0.017	0.001	0.000	0.000	-0.001	-0.002	0.000	0.000	0.005	0.022	-0.012
40-44	0.031	0.002	-0.017	0.000	0.001	0.000	-0.001	-0.001	0.000	0.000	0.003	0.045	-0.014
45-49	0.065	0.002	-0.013	0.000	0.001	0.000	-0.001	-0.003	0.000	0.000	0.002	0.076	-0.011
50-54	0.089	0.001	-0.010	0.001	0.000	0.000	-0.001	-0.002	0.000	0.000	0.002	0.096	-0.007
55-59	0.136	0.001	-0.006	0.000	0.000	0.000	-0.002	0.000	0.000	0.000	0.001	0.141	-0.005
60-64	0.185	0.001	-0.003	-0.001	0.000	0.000	-0.002	0.000	0.000	0.000	0.001	0.188	-0.004
65-69	0.276	0.001	-0.002	-0.002	0.000	0.000	-0.002	0.000	0.000	0.000	0.001	0.279	-0.003
70-74	0.386	0.001	0.000	-0.002	0.000	0.000	-0.001	0.000	0.000	0.000	0.001	0.388	-0.002
75-79	0.428	0.001	0.000	-0.003	0.000	0.000	0.001	0.000	0.000	0.000	0.001	0.428	-0.001
80-84	0.415	0.001	0.000	-0.006	0.000	0.000	0.003	0.000	0.000	0.000	0.001	0.416	-0.001
≥85	0.394	0.001	0.000	-0.025	0.001	0.000	0.015	0.000	0.000	0.000	0.001	0.401	-0.008
Total	**2.681**	**0.052**	**-0.089**	**-0.035**	**0.009**	**0.002**	**0.001**	**-0.020**	**-0.001**	**0.000**	**0.048**	**2.714**	**-0.033**

Table A.8: Contributions of injury deaths to changes in LE for females in the US

Age groups	Age contribution	Transport	Poison	Fall	Fire	Drowning	Other unintentional	Self-harm	Assault	Other intentional	Undetermined intent	Non injury	Total Injury
<1	0.100	0.001	0.000	0.000	0.001	0.000	0.001	0.000	0.001	0.000	-0.001	0.096	0.004
1-4	0.023	0.004	0.000	0.000	0.002	0.001	0.001	0.000	0.002	0.000	0.000	0.014	0.010
5-9	0.011	0.005	0.000	0.000	0.001	0.000	0.000	0.000	0.000	0.000	0.000	0.004	0.007
10-14	0.010	0.007	0.000	0.000	0.001	0.000	0.000	-0.004	0.000	0.000	0.000	0.006	0.004
15-19	0.032	0.031	-0.003	0.000	0.000	0.000	0.000	-0.008	0.001	0.000	0.000	0.010	0.022
20-24	-0.002	0.012	-0.022	0.000	0.001	0.000	0.000	-0.008	0.002	0.000	0.000	0.013	-0.015
25-29	-0.033	0.003	-0.038	0.000	0.001	0.000	0.000	-0.007	0.002	0.000	-0.001	0.007	-0.040
30-34	-0.046	0.002	-0.042	0.000	0.001	0.000	0.000	-0.006	0.001	0.000	-0.001	0.000	-0.046
35-39	-0.016	0.005	-0.035	0.000	0.000	0.000	0.000	-0.005	0.002	0.000	0.001	0.016	-0.032
40-44	0.033	0.005	-0.023	0.000	0.001	0.000	0.000	-0.003	0.002	0.000	0.002	0.050	-0.017
45-49	0.045	0.003	-0.021	0.000	0.000	0.000	0.000	-0.004	0.001	0.000	0.001	0.066	-0.021
50-54	0.029	0.003	-0.024	-0.001	0.000	0.000	0.000	-0.005	0.000	0.000	-0.001	0.057	-0.028
55-59	0.058	0.002	-0.020	-0.001	0.000	0.000	-0.001	-0.004	0.000	0.000	-0.001	0.084	-0.026
60-64	0.146	0.003	-0.010	-0.002	0.000	0.000	-0.001	-0.003	0.000	0.000	-0.001	0.160	-0.014
65-69	0.238	0.003	-0.004	-0.002	0.000	0.000	-0.001	-0.002	0.000	0.000	0.000	0.244	-0.006
70-74	0.243	0.003	-0.001	-0.004	0.000	0.000	0.000	-0.001	0.000	0.000	0.000	0.247	-0.004
75-79	0.253	0.002	0.000	-0.006	0.000	0.000	0.001	0.000	0.000	0.000	0.000	0.256	-0.003
80-84	0.309	0.002	0.000	-0.007	0.000	0.000	0.003	0.000	0.000	0.000	0.000	0.311	-0.002
≥85	0.336	0.001	0.000	-0.032	0.000	0.000	0.009	0.000	0.000	0.000	0.000	0.358	-0.022
Total	**1.769**	**0.096**	**-0.242**	**-0.055**	**0.009**	**-0.001**	**0.012**	**-0.061**	**0.015**	**0.000**	**-0.002**	**1.998**	**-0.229**

Table A.9: Contributions of injury deaths to changes in LE for females in all G7 countries combined

Age groups	Age contribution	Transport	Poison	Fall	Fire	Drowning	Other unintentional	Self-harm	Assault	Other intentional	Undetermined intent	Non injury	Total Injury
<1	0.085	0.001	0.000	0.000	0.000	0.000	0.001	0.000	0.001	0.000	0.000	0.080	0.004
1-4	0.024	0.004	0.000	0.000	0.001	0.001	0.001	0.000	0.001	0.000	0.000	0.015	0.009
5-9	0.012	0.004	0.000	0.000	0.001	0.000	0.000	0.000	0.000	0.000	0.000	0.005	0.006
10-14	0.010	0.006	0.000	0.000	0.000	0.000	0.000	-0.003	0.000	0.000	0.000	0.006	0.004
15-19	0.028	0.023	-0.002	0.000	0.000	0.000	0.000	-0.005	0.000	0.000	0.001	0.010	0.017
20-24	0.008	0.012	-0.013	0.000	0.001	0.000	0.000	-0.004	0.001	0.000	0.000	0.011	-0.002
25-29	-0.013	0.004	-0.021	0.000	0.000	0.000	0.000	-0.002	0.000	0.000	0.000	0.005	-0.019
30-34	-0.016	0.002	-0.023	0.000	0.000	0.000	0.000	-0.001	0.000	0.000	0.000	0.004	-0.020
35-39	0.009	0.004	-0.019	0.000	0.000	0.000	0.000	0.000	0.001	0.000	0.001	0.021	-0.012
40-44	0.054	0.005	-0.011	0.000	0.000	0.000	0.000	0.001	0.001	0.000	0.001	0.056	-0.002
45-49	0.069	0.004	-0.011	0.000	0.000	0.000	0.000	0.000	0.001	0.000	0.001	0.074	-0.005
50-54	0.056	0.003	-0.012	0.000	0.000	0.000	0.000	0.000	0.000	0.000	0.000	0.065	-0.009
55-59	0.053	0.002	-0.011	-0.001	0.000	0.000	-0.001	0.001	0.000	0.000	0.000	0.061	-0.008
60-64	0.071	0.003	-0.006	-0.001	0.000	0.000	-0.001	0.002	0.000	0.000	0.000	0.074	-0.003
65-69	0.167	0.003	-0.002	-0.001	0.000	0.000	-0.001	0.002	0.000	0.000	0.000	0.165	0.002
70-74	0.232	0.003	0.000	-0.002	0.000	0.000	0.000	0.001	0.000	0.000	0.000	0.230	0.002
75-79	0.341	0.003	0.000	-0.003	0.000	0.000	0.002	0.001	0.000	0.000	0.000	0.337	0.003
80-84	0.416	0.002	0.000	-0.004	0.000	-0.001	0.004	0.001	0.000	0.000	0.000	0.412	0.004
≥85	0.520	0.002	0.000	-0.022	0.000	-0.001	0.013	0.002	0.000	0.000	0.001	0.525	-0.006
Total	**2.124**	**0.088**	**-0.130**	**-0.032**	**0.008**	**0.002**	**0.019**	**-0.003**	**0.008**	**0.000**	**0.005**	**2.158**	**-0.034**

Table A.10: Contributions of injury deaths to changes in LE for males in Canada

Age groups	Age contribution	Transport	Poison	Fall	Fire	Drowning	Other unintentional	Self-harm	Assault	Other intentional	Undetermined intent	Non injury	Total Injury
<1	0.071	0.001	0.000	0.000	0.000	0.001	0.002	0.000	0.001	0.000	0.000	0.068	0.003
1-4	0.021	0.003	0.000	0.000	0.002	0.002	0.001	0.000	0.002	0.000	0.000	0.011	0.011
5-9	0.016	0.006	0.000	0.000	0.001	0.001	0.000	0.000	0.000	0.000	0.000	0.007	0.009
10-14	0.018	0.011	0.000	0.000	0.001	0.001	0.001	0.000	0.001	0.000	0.000	0.004	0.014
15-19	0.057	0.049	-0.010	0.001	0.000	0.002	0.002	0.002	-0.001	0.000	0.002	0.009	0.048
20-24	0.019	0.042	-0.040	0.003	0.001	0.001	0.004	0.003	0.001	0.000	0.002	0.003	0.017
25-29	-0.047	0.022	-0.063	0.000	0.001	0.000	0.002	-0.002	-0.001	0.000	0.003	-0.009	-0.038
30-34	-0.043	0.010	-0.061	0.000	0.001	0.000	0.001	0.002	-0.002	0.000	0.002	0.003	-0.046
35-39	-0.015	0.012	-0.056	0.000	0.001	0.000	0.002	0.008	0.000	0.000	0.004	0.013	-0.027
40-44	0.035	0.010	-0.037	0.002	0.001	0.001	0.002	0.006	0.001	0.000	0.005	0.044	-0.009
45-49	0.091	0.010	-0.034	0.002	0.000	0.001	0.003	0.004	-0.001	0.000	0.004	0.102	-0.012
50-54	0.125	0.006	-0.030	0.000	0.000	0.000	0.001	0.003	0.000	0.000	0.003	0.143	-0.017
55-59	0.178	0.004	-0.023	-0.001	0.000	0.000	0.000	-0.002	0.000	0.000	0.001	0.198	-0.020
60-64	0.263	0.003	-0.013	-0.002	0.000	0.000	-0.001	-0.003	0.000	0.000	0.000	0.278	-0.015
65-69	0.354	0.003	-0.005	-0.002	0.000	0.000	0.002	-0.001	0.000	0.000	0.001	0.355	-0.001
70-74	0.445	0.003	-0.001	-0.005	0.000	0.000	0.002	0.001	0.000	0.000	0.000	0.444	0.001
75-79	0.490	0.002	0.000	-0.008	0.000	0.000	0.005	0.000	0.000	0.000	0.000	0.490	0.000
80-84	0.487	0.003	0.000	-0.008	0.000	0.000	0.006	0.000	0.000	0.000	0.000	0.486	0.001
≥85	0.490	0.002	0.000	-0.033	0.000	0.000	0.021	0.001	0.000	0.000	0.000	0.499	-0.009
Total	**3.056**	**0.203**	**-0.372**	**-0.050**	**0.010**	**0.011**	**0.055**	**0.023**	**0.001**	**-0.001**	**0.029**	**3.147**	**-0.091**

Table A.11: Contributions of injury deaths to changes in LE for males in France

Age groups	Age contribution	Transport	Poison	Fall	Fire	Drowning	Other unintentional	Self-harm	Assault	Other intentional	Undetermined intent	Non injury	Total Injury
<1	0.052	0.001	0.000	0.000	0.000	0.001	0.003	0.000	0.001	0.000	0.000	0.047	0.006
1-4	0.031	0.005	0.000	0.001	0.001	0.005	0.002	0.000	0.001	0.000	0.000	0.016	0.015
5-9	0.020	0.005	0.000	0.000	0.001	0.002	0.002	0.000	0.001	0.000	0.000	0.010	0.011
10-14	0.026	0.010	0.000	0.001	0.000	0.001	0.001	0.002	0.001	0.000	0.000	0.011	0.015
15-19	0.098	0.067	0.000	0.001	0.000	0.001	0.002	0.009	0.000	0.000	0.000	0.018	0.080
20-24	0.125	0.078	0.001	0.002	0.001	0.002	0.002	0.019	0.000	0.000	-0.002	0.022	0.102
25-29	0.096	0.050	0.000	0.001	0.001	0.003	0.001	0.022	-0.001	0.000	-0.002	0.021	0.075
30-34	0.093	0.030	-0.001	0.002	0.001	0.002	0.002	0.027	0.000	0.000	-0.001	0.030	0.062
35-39	0.127	0.024	-0.002	0.003	0.001	0.002	0.003	0.027	0.001	0.000	-0.001	0.070	0.057
40-44	0.210	0.019	-0.003	0.004	0.001	0.003	0.004	0.029	0.001	0.000	-0.001	0.153	0.056
45-49	0.298	0.016	-0.003	0.004	0.001	0.003	0.003	0.017	0.000	0.000	-0.001	0.256	0.042
50-54	0.296	0.011	-0.002	0.004	0.000	0.003	0.003	0.003	0.000	0.000	-0.002	0.276	0.019
55-59	0.289	0.008	-0.002	0.002	0.000	0.002	-0.002	0.002	0.000	0.000	-0.003	0.280	0.008
60-64	0.194	0.006	-0.001	0.001	0.000	0.001	0.000	0.003	0.000	0.000	-0.002	0.186	0.008
65-69	0.315	0.007	-0.001	0.001	0.000	0.001	0.001	0.006	0.000	0.000	-0.001	0.301	0.014
70-74	0.435	0.006	0.000	0.001	0.000	0.001	0.002	0.007	0.000	0.000	-0.001	0.420	0.016
75-79	0.495	0.004	0.000	0.001	0.000	0.000	0.006	0.006	0.000	0.000	-0.001	0.478	0.017
80-84	0.512	0.004	0.000	0.000	0.000	0.000	0.010	0.005	0.000	0.000	-0.001	0.493	0.019
≥85	0.466	0.002	-0.001	-0.009	0.000	0.000	0.022	0.006	0.000	0.000	-0.001	0.445	0.021
Total	**4.177**	**0.351**	**-0.012**	**0.020**	**0.011**	**0.033**	**0.065**	**0.189**	**0.005**	**0.000**	**-0.019**	**3.535**	**0.642**

Table A.12: Contributions of injury deaths to changes in LE for males in Germany

Age groups	Age contribution	Transport	Poison	Fall	Fire	Drowning	Other unintentional	Self-harm	Assault	Other intentional	Undetermined intent	Non injury	Total Injury
<1	0.082	0.001	0.000	0.001	0.000	0.000	0.001	0.000	0.001	0.000	0.001	0.077	0.005
1-4	0.025	0.003	0.000	0.000	0.002	0.004	0.000	0.000	0.000	0.000	0.001	0.014	0.011
5-9	0.013	0.005	0.000	0.000	0.001	0.002	0.000	0.000	0.000	0.000	0.000	0.006	0.008
10-14	0.019	0.006	0.000	0.001	0.000	0.001	0.001	0.001	0.000	0.000	0.001	0.008	0.011
15-19	0.087	0.058	0.000	0.002	0.001	0.000	0.000	0.009	0.001	0.000	0.003	0.014	0.073
20-24	0.111	0.064	0.003	0.001	0.001	0.000	0.000	0.014	0.001	0.000	0.003	0.023	0.088
25-29	0.073	0.029	0.004	0.001	0.001	-0.001	0.001	0.011	0.002	0.000	0.003	0.022	0.051
30-34	0.049	0.016	-0.002	0.001	0.000	0.000	0.001	0.011	0.001	0.000	0.001	0.019	0.030
35-39	0.077	0.015	-0.003	0.001	0.001	0.000	0.001	0.014	0.001	0.000	0.002	0.045	0.032
40-44	0.151	0.012	-0.003	0.002	0.001	0.001	0.000	0.018	0.000	0.000	0.002	0.117	0.033
45-49	0.240	0.010	-0.002	0.003	0.001	0.001	0.001	0.015	0.001	0.000	0.003	0.206	0.033
50-54	0.243	0.005	-0.002	0.002	0.001	0.001	0.000	0.009	0.001	0.000	0.002	0.224	0.019
55-59	0.187	0.002	-0.001	0.000	0.000	0.000	-0.003	0.004	0.000	0.000	0.001	0.184	0.003
60-64	0.153	0.002	-0.001	-0.002	0.000	0.000	-0.004	0.003	0.000	0.000	0.001	0.154	-0.001
65-69	0.288	0.002	0.000	-0.003	0.000	0.000	-0.003	0.003	0.000	0.000	0.000	0.288	0.000
70-74	0.425	0.002	0.000	-0.003	0.000	0.000	-0.004	0.004	0.000	0.000	0.000	0.426	0.000
75-79	0.466	0.002	0.000	-0.005	0.000	0.000	-0.004	0.003	0.000	0.000	0.000	0.470	-0.004
80-84	0.373	0.001	0.000	-0.006	0.000	0.000	-0.004	0.003	0.000	0.000	0.001	0.378	-0.005
≥85	0.276	0.000	0.000	-0.017	0.000	0.000	-0.007	0.003	0.000	0.000	0.001	0.295	-0.020
Total	**3.338**	**0.237**	**-0.007**	**-0.022**	**0.010**	**0.009**	**-0.021**	**0.125**	**0.011**	**0.000**	**0.026**	**2.972**	**0.367**

Table A.13: Contributions of injury deaths to changes in LE for males in Italy

Age groups	Age contribution	Transport	Poison	Fall	Fire	Drowning	Other unintentional	Self-harm	Assault	Other intentional	Undetermined intent	Non injury	Total Injury
<1	0.093	0.001	0.000	0.000	0.000	0.000	0.001	0.000	0.000	0.000	0.000	0.090	0.003
1-4	0.013	0.003	0.000	0.001	0.000	0.000	0.000	0.000	-0.001	0.000	0.000	0.010	0.003
5-9	0.012	0.002	0.000	0.000	0.000	0.000	0.001	0.000	0.000	0.000	0.000	0.009	0.003
10-14	0.018	0.009	0.000	0.000	0.000	0.000	0.002	0.000	0.000	0.000	0.000	0.006	0.011
15-19	0.072	0.051	0.000	0.002	0.000	-0.002	0.004	0.000	0.002	0.000	0.000	0.015	0.057
20-24	0.100	0.070	-0.001	0.002	0.000	0.001	0.007	0.000	0.005	0.000	0.000	0.016	0.084
25-29	0.090	0.051	-0.001	0.002	0.000	0.000	0.004	0.005	0.006	0.000	0.000	0.023	0.067
30-34	0.071	0.031	-0.002	0.001	0.000	0.000	0.004	0.002	0.004	0.000	0.000	0.030	0.041
35-39	0.082	0.017	-0.003	0.001	0.000	0.001	0.003	0.003	0.003	0.000	0.001	0.055	0.027
40-44	0.095	0.012	-0.002	0.001	0.000	0.001	0.001	0.002	0.002	0.000	0.000	0.076	0.019
45-49	0.109	0.011	-0.002	0.002	0.000	0.000	0.001	0.001	0.001	0.000	0.000	0.096	0.014
50-54	0.141	0.006	-0.002	0.001	0.000	0.000	0.000	-0.001	0.001	0.000	0.000	0.134	0.006
55-59	0.194	0.003	-0.001	0.002	0.000	0.000	-0.001	-0.002	0.001	0.000	0.000	0.191	0.003
60-64	0.243	0.003	0.000	0.001	0.000	0.000	0.000	0.000	0.000	0.000	0.000	0.239	0.004
65-69	0.331	0.004	0.000	0.001	0.000	0.000	-0.001	0.001	0.000	0.000	0.000	0.325	0.005
70-74	0.408	0.004	0.000	0.001	0.000	0.000	0.000	0.003	0.000	0.000	0.000	0.400	0.008
75-79	0.480	0.004	0.000	0.000	0.000	0.000	0.003	0.002	0.000	0.000	0.000	0.470	0.010
80-84	0.388	0.003	0.000	-0.001	0.000	0.000	0.003	0.002	0.000	0.000	0.000	0.381	0.007
≥85	0.323	0.001	0.000	-0.004	0.000	0.000	0.006	0.001	0.000	0.000	0.000	0.318	0.004
Total	**3.262**	**0.286**	**-0.013**	**0.013**	**0.001**	**0.003**	**0.039**	**0.020**	**0.027**	**0.000**	**0.004**	**2.884**	**0.378**

Table A.14: Contributions of injury deaths to changes in LE for males in Japan

Age groups	Age contribution	Transport	Poison	Fall	Fire	Drowning	Other unintentional	Self-harm	Assault	Other intentional	Undetermined intent	Non injury	Total Injury
<1	0.101	0.001	0.000	0.001	0.000	0.000	0.004	0.000	0.002	0.000	-0.001	0.095	0.006
1-4	0.034	0.006	0.000	0.002	0.002	0.005	0.002	0.000	0.002	0.000	0.000	0.017	0.017
5-9	0.019	0.007	0.000	0.000	0.001	0.002	0.000	0.000	0.001	0.000	0.000	0.006	0.013
10-14	0.008	0.004	0.000	0.000	0.001	0.001	0.001	-0.004	0.000	0.000	-0.001	0.006	0.003
15-19	0.050	0.038	0.000	0.001	0.001	0.001	0.001	-0.010	0.001	0.000	0.000	0.018	0.033
20-24	0.039	0.031	0.000	0.002	0.001	0.002	0.001	-0.012	0.001	0.000	-0.001	0.014	0.025
25-29	0.048	0.022	0.001	0.002	0.001	0.001	0.001	0.004	0.001	0.000	0.001	0.015	0.033
30-34	0.060	0.017	0.001	0.002	0.001	0.001	0.001	0.009	0.001	0.000	0.001	0.027	0.033
35-39	0.085	0.013	0.000	0.001	0.001	0.001	0.001	0.019	0.001	0.000	0.002	0.046	0.039
40-44	0.122	0.011	0.001	0.001	0.001	0.002	0.001	0.035	0.001	0.000	0.001	0.068	0.054
45-49	0.147	0.010	0.000	0.002	0.001	0.002	0.002	0.034	0.001	0.000	0.001	0.094	0.053
50-54	0.221	0.010	0.000	0.003	0.001	0.002	0.002	0.041	0.001	0.000	0.001	0.159	0.062
55-59	0.273	0.010	0.000	0.003	0.002	0.003	0.000	0.046	0.001	0.000	0.001	0.207	0.065
60-64	0.251	0.010	0.000	0.003	0.001	0.001	0.001	0.030	0.001	0.000	0.000	0.203	0.048
65-69	0.301	0.009	0.000	0.003	0.001	0.001	0.001	0.018	0.000	0.000	0.000	0.268	0.033
70-74	0.360	0.009	0.000	0.003	0.001	0.000	0.002	0.007	0.000	0.000	-0.001	0.339	0.021
75-79	0.417	0.008	0.000	0.002	0.001	0.000	0.004	0.004	0.000	0.000	-0.001	0.399	0.018
80-84	0.363	0.006	0.000	0.001	0.001	0.000	0.004	0.004	0.000	0.000	-0.001	0.348	0.015
≥85	0.267	0.005	0.000	-0.005	0.001	-0.001	0.003	0.006	0.000	0.000	-0.001	0.258	0.010
Total	**3.168**	**0.225**	**0.005**	**0.029**	**0.019**	**0.026**	**0.030**	**0.232**	**0.014**	**0.000**	**0.000**	**2.587**	**0.581**

Table A.15: Contributions of injury deaths to changes in LE for males in the UK

Age groups	Age contribution	Transport	Poison	Fall	Fire	Drowning	Other unintentional	Self-harm	Assault	Other intentional	Undetermined intent	Non injury	Total Injury
<1	0.143	0.000	0.000	0.000	0.000	0.000	-0.001	0.000	0.000	0.000	0.004	0.138	0.004
1-4	0.035	0.003	0.000	0.000	0.003	0.001	-0.001	0.000	0.000	0.000	0.003	0.025	0.009
5-9	0.016	0.003	0.000	0.000	0.001	0.001	0.000	0.000	0.000	0.000	0.001	0.009	0.007
10-14	0.021	0.008	0.000	0.001	0.000	0.000	-0.001	-0.001	0.000	0.000	0.001	0.013	0.008
15-19	0.075	0.039	-0.002	0.001	0.001	0.000	-0.004	-0.004	0.000	0.000	0.014	0.030	0.045
20-24	0.097	0.038	-0.006	0.002	0.001	0.000	-0.005	0.000	0.000	0.000	0.020	0.048	0.049
25-29	0.072	0.024	-0.014	0.002	0.001	0.000	-0.003	0.003	0.000	0.000	0.016	0.043	0.028
30-34	0.058	0.019	-0.029	0.002	0.001	0.000	-0.002	0.003	-0.001	0.000	0.016	0.050	0.008
35-39	0.029	0.013	-0.040	0.001	0.001	0.000	-0.002	0.001	0.000	0.000	0.012	0.043	-0.014
40-44	0.034	0.009	-0.045	0.002	0.001	0.000	-0.002	-0.003	0.000	0.000	0.007	0.066	-0.032
45-49	0.070	0.006	-0.035	0.002	0.001	0.000	-0.003	-0.008	-0.001	0.000	0.006	0.101	-0.031
50-54	0.118	0.004	-0.019	0.001	0.000	0.000	-0.002	-0.008	-0.001	0.000	0.004	0.138	-0.021
55-59	0.218	0.003	-0.009	0.000	0.000	0.000	-0.002	-0.004	0.000	0.000	0.003	0.227	-0.009
60-64	0.327	0.002	-0.005	0.000	0.000	0.000	-0.003	-0.003	0.000	0.000	0.002	0.335	-0.008
65-69	0.432	0.002	-0.002	-0.002	0.000	0.000	-0.003	0.000	0.000	0.000	0.002	0.435	-0.003
70-74	0.556	0.002	-0.001	-0.002	0.000	0.000	-0.002	0.000	0.000	0.000	0.001	0.558	-0.001
75-79	0.560	0.001	0.000	-0.004	0.000	0.000	-0.001	0.000	0.000	0.000	0.001	0.563	-0.003
80-84	0.465	0.001	0.000	-0.005	0.000	0.000	0.001	0.000	0.000	0.000	0.000	0.468	-0.003
≥85	0.368	0.001	0.000	-0.017	0.000	0.000	0.003	0.000	0.000	0.000	0.000	0.380	-0.012
Total	**3.692**	**0.179**	**-0.207**	**-0.016**	**0.013**	**0.003**	**-0.035**	**-0.026**	**-0.001**	**0.000**	**0.113**	**3.670**	**0.022**

Table A.16: Contributions of injury deaths to changes in LE for males in the US

Age groups	Age contribution	Transport	Poison	Fall	Fire	Drowning	Other unintentional	Self-harm	Assault	Other intentional	Undetermined intent	Non injury	Total Injury
<1	0.122	0.001	0.000	0.000	0.000	0.000	0.001	0.000	0.001	0.000	-0.001	0.118	0.004
1-4	0.027	0.006	0.000	0.001	0.003	0.001	0.001	0.000	0.002	0.000	-0.001	0.014	0.014
5-9	0.014	0.007	0.000	0.000	0.001	0.001	0.000	0.000	0.000	0.000	0.000	0.005	0.009
10-14	0.016	0.010	0.000	0.000	0.001	0.001	0.001	-0.005	0.000	0.000	0.000	0.008	0.009
15-19	0.065	0.058	-0.003	0.001	0.001	0.003	0.002	-0.014	0.003	0.000	0.001	0.015	0.050
20-24	0.029	0.045	-0.040	0.001	0.001	0.002	0.002	-0.018	0.017	0.000	0.001	0.017	0.012
25-29	-0.072	0.014	-0.083	0.001	0.001	0.001	0.001	-0.017	0.004	-0.001	-0.001	0.008	-0.080
30-34	-0.092	0.007	-0.091	0.000	0.001	0.000	0.001	-0.015	-0.004	0.000	-0.001	0.011	-0.103
35-39	-0.045	0.006	-0.077	0.000	0.001	0.000	0.001	-0.011	-0.003	0.000	0.001	0.038	-0.083
40-44	0.048	0.009	-0.050	0.001	0.001	0.000	0.001	-0.006	-0.002	0.000	0.002	0.091	-0.043
45-49	0.108	0.007	-0.039	0.001	0.001	0.000	0.001	-0.007	0.001	0.000	0.002	0.143	-0.035
50-54	0.081	0.001	-0.041	-0.001	0.001	0.000	-0.001	-0.007	0.000	0.000	-0.001	0.131	-0.050
55-59	0.061	-0.001	-0.036	-0.003	0.000	0.000	-0.002	-0.009	-0.001	0.000	-0.002	0.114	-0.053
60-64	0.123	0.000	-0.021	-0.003	0.000	0.000	-0.002	-0.007	0.000	0.000	-0.001	0.158	-0.035
65-69	0.240	0.001	-0.009	-0.004	0.000	0.000	-0.001	-0.003	0.000	0.000	-0.001	0.256	-0.016
70-74	0.309	0.001	-0.002	-0.005	0.000	0.000	-0.001	-0.001	0.000	0.000	0.000	0.316	-0.007
75-79	0.316	0.002	0.000	-0.006	0.000	0.000	0.001	-0.001	0.000	0.000	0.000	0.320	-0.004
80-84	0.334	0.002	0.000	-0.006	0.000	0.000	0.002	0.001	0.000	0.000	0.000	0.334	0.000
≥85	0.327	0.002	0.000	-0.016	0.000	0.000	0.005	0.000	0.000	0.000	0.000	0.334	-0.007
Total	**2.011**	**0.178**	**-0.492**	**-0.036**	**0.014**	**0.008**	**0.014**	**-0.118**	**0.018**	**-0.003**	**-0.003**	**2.431**	**-0.420**

Table A.17: Contributions of injury deaths to changes in LE for males in all G7 countries combined

Age groups	Age contribution	Transport	Poison	Fall	Fire	Drowning	Other unintentional	Self-harm	Assault	Other intentional	Undetermined intent	Non injury	Total Injury
<1	0.102	0.001	0.000	0.000	0.000	0.000	0.002	0.000	0.001	0.000	0.000	0.097	0.004
1-4	0.028	0.005	0.000	0.001	0.002	0.002	0.001	0.000	0.001	0.000	0.000	0.015	0.012
5-9	0.015	0.006	0.000	0.000	0.001	0.001	0.000	0.000	0.000	0.000	0.000	0.006	0.009
10-14	0.017	0.009	0.000	0.000	0.000	0.001	0.001	-0.003	0.000	0.000	0.000	0.008	0.009
15-19	0.066	0.053	-0.002	0.001	0.001	0.001	0.001	-0.007	0.001	0.000	0.002	0.016	0.050
20-24	0.047	0.046	-0.023	0.001	0.001	0.002	0.002	-0.007	0.006	0.000	0.002	0.018	0.029
25-29	-0.018	0.020	-0.048	0.001	0.001	0.001	0.001	-0.004	-0.002	0.000	0.001	0.011	-0.029
30-34	-0.023	0.013	-0.051	0.001	0.001	0.000	0.001	-0.002	-0.003	0.000	0.001	0.017	-0.040
35-39	0.014	0.011	-0.043	0.001	0.001	0.001	0.001	0.001	-0.002	0.000	0.002	0.041	-0.027
40-44	0.096	0.012	-0.026	0.002	0.001	0.001	0.001	0.007	0.000	0.000	0.002	0.096	0.000
45-49	0.150	0.009	-0.021	0.002	0.001	0.001	0.001	0.006	0.001	0.000	0.002	0.148	0.002
50-54	0.145	0.004	-0.021	0.001	0.001	0.001	0.000	0.007	0.000	0.000	0.000	0.152	-0.007
55-59	0.135	0.002	-0.019	0.000	0.000	0.001	-0.001	0.005	0.000	0.000	0.000	0.148	-0.013
60-64	0.156	0.002	-0.011	-0.001	0.000	0.000	-0.001	0.004	0.000	0.000	0.000	0.165	-0.008
65-69	0.280	0.003	-0.004	-0.001	0.000	0.000	-0.001	0.004	0.000	0.000	0.000	0.279	0.001
70-74	0.375	0.003	-0.001	-0.002	0.000	0.000	0.000	0.002	0.000	0.000	0.000	0.372	0.003
75-79	0.415	0.003	0.000	-0.003	0.000	0.000	0.001	0.002	0.000	0.000	0.000	0.412	0.003
80-84	0.388	0.003	0.000	-0.004	0.000	0.000	0.002	0.002	0.000	0.000	0.000	0.385	0.003
≥85	0.343	0.002	0.000	-0.013	0.000	0.000	0.004	0.002	0.000	0.000	0.000	0.346	-0.004
Total	**2.732**	**0.207**	**-0.268**	**-0.012**	**0.013**	**0.012**	**0.015**	**0.019**	**0.004**	**-0.002**	**0.010**	**2.735**	**-0.003**

Table A.18: Contributions of injury deaths to changes in LD for females in Canada

Age groups	Age contribution	Transport	Poison	Fall	Fire	Drowning	Other unintentional	Self-harm	Assault	Other intentional	Undetermined intent	Non injury	Total Injury
<1	-0.036	-0.001	0.000	0.000	0.000	0.000	-0.001	0.000	-0.001	0.000	0.000	-0.034	-0.002
1-4	-0.016	-0.002	0.000	0.000	-0.001	-0.001	-0.001	0.000	-0.002	0.000	0.000	-0.009	-0.007
5-9	-0.006	-0.004	0.000	0.000	-0.001	0.000	-0.001	0.000	0.000	0.000	0.000	-0.001	-0.005
10-14	-0.008	-0.006	0.000	0.000	0.000	0.000	0.000	0.004	0.000	0.000	0.000	-0.005	-0.002
15-19	-0.013	-0.014	0.006	0.000	0.000	-0.001	-0.001	0.006	-0.001	0.000	0.000	-0.007	-0.006
20-24	0.006	-0.010	0.014	0.000	0.000	0.000	0.000	0.007	-0.001	0.000	0.000	-0.003	0.009
25-29	0.021	-0.002	0.018	0.000	-0.001	0.000	0.001	0.006	0.000	0.000	-0.001	0.001	0.020
30-34	0.013	-0.003	0.018	0.000	0.000	0.000	0.000	-0.001	0.000	0.000	-0.002	0.000	0.013
35-39	-0.008	-0.004	0.013	0.000	0.000	0.000	-0.001	-0.002	0.000	0.000	-0.001	-0.014	0.006
40-44	-0.029	-0.004	0.010	0.000	0.000	-0.001	0.000	-0.001	-0.001	0.000	-0.001	-0.031	0.002
45-49	-0.048	-0.003	0.009	0.000	0.000	0.000	0.000	-0.001	0.000	0.000	-0.001	-0.052	0.004
50-54	-0.057	-0.002	0.009	0.000	0.000	0.000	0.000	0.000	0.000	0.000	-0.001	-0.064	0.007
55-59	-0.068	-0.002	0.006	0.001	0.000	0.000	0.000	0.001	0.000	0.000	-0.001	-0.072	0.004
60-64	-0.090	-0.001	0.002	0.001	0.000	0.000	0.000	0.001	0.000	0.000	-0.001	-0.093	0.002
65-69	-0.099	-0.001	0.001	0.002	0.000	0.000	0.000	0.000	0.000	0.000	0.000	-0.100	0.001
70-74	-0.074	-0.001	0.000	0.001	0.000	0.000	-0.001	0.000	0.000	0.000	0.000	-0.074	0.000
75-79	-0.031	0.000	0.000	0.001	0.000	0.000	-0.001	0.000	0.000	0.000	0.000	-0.031	0.000
80-84	0.134	0.001	0.000	-0.005	0.000	0.000	0.003	0.000	0.000	0.000	0.000	0.135	-0.002
≥85	0.613	0.001	0.000	-0.081	0.001	0.000	0.048	0.000	0.000	0.000	0.000	0.643	-0.031
Total	**0.202**	**-0.059**	**0.107**	**-0.080**	**-0.003**	**0.000**	**0.045**	**0.019**	**-0.006**	**0.000**	**-0.009**	**0.188**	**0.014**

Table A.19: Contributions of injury deaths to changes in LD for females in France

Age groups	Age contribution	Transport	Poison	Fall	Fire	Drowning	Other unintentional	Self-harm	Assault	Other intentional	Undetermined intent	Non injury	Total Injury
<1	-0.026	-0.001	0.000	0.000	0.000	0.000	-0.001	0.000	0.000	0.000	0.000	-0.023	-0.003
1-4	-0.025	-0.004	0.000	-0.001	-0.001	-0.002	-0.001	0.000	-0.001	0.000	0.000	-0.014	-0.011
5-9	-0.013	-0.003	0.000	0.000	0.000	-0.001	0.000	0.000	0.000	0.000	0.000	-0.008	-0.005
10-14	-0.015	-0.006	0.000	0.000	0.000	0.000	0.000	0.000	0.000	0.000	0.000	-0.008	-0.007
15-19	-0.033	-0.019	0.000	0.000	0.000	0.000	0.000	0.000	-0.001	0.000	0.000	-0.012	-0.021
20-24	-0.035	-0.017	0.000	0.000	0.000	0.000	-0.001	-0.003	-0.001	0.000	0.000	-0.012	-0.023
25-29	-0.028	-0.010	0.000	0.000	0.000	0.000	-0.001	-0.003	-0.001	0.000	-0.001	-0.012	-0.016
30-34	-0.034	-0.008	0.000	0.000	0.000	0.000	0.000	-0.007	-0.001	0.000	0.000	-0.016	-0.018
35-39	-0.054	-0.006	0.000	-0.001	-0.001	-0.001	0.000	-0.009	-0.001	0.000	0.000	-0.036	-0.017
40-44	-0.079	-0.006	0.000	-0.001	-0.001	0.000	-0.002	-0.011	-0.001	0.000	0.000	-0.059	-0.021
45-49	-0.083	-0.004	0.001	-0.001	0.000	-0.001	-0.002	-0.007	0.000	0.000	0.000	-0.067	-0.016
50-54	-0.055	-0.004	0.000	-0.001	0.000	-0.001	-0.001	-0.005	-0.001	0.000	0.000	-0.045	-0.011
55-59	-0.046	-0.003	0.001	-0.001	0.000	-0.001	-0.001	-0.004	0.000	0.000	0.000	-0.037	-0.009
60-64	-0.021	-0.003	0.000	-0.001	0.000	-0.001	0.000	-0.003	0.000	0.000	0.000	-0.014	-0.007
65-69	-0.056	-0.002	0.000	0.000	0.000	0.000	-0.001	-0.002	0.000	0.000	0.000	-0.051	-0.005
70-74	-0.082	-0.002	0.000	0.000	0.000	0.000	-0.002	-0.001	0.000	0.000	0.000	-0.077	-0.005
75-79	-0.074	-0.001	0.000	0.000	0.000	0.000	-0.002	0.000	0.000	0.000	0.000	-0.070	-0.004
80-84	0.124	0.001	0.000	0.001	0.000	0.000	0.005	0.000	0.000	0.000	0.000	0.117	0.007
≥85	1.043	0.002	0.0011	-0.016	0.001	0.000	0.089	0.002	0.000	0.000	-0.001	0.965	0.077
Total	**0.408**	**-0.096**	**0.003**	**-0.023**	**-0.004**	**-0.007**	**0.080**	**-0.055**	**-0.009**	**0.000**	**0.000**	**0.520**	**-0.117**

Table A.20: Contributions of injury deaths to changes in LD for females in Germany

Age groups	Age contribution	Transport	Poison	Fall	Fire	Drowning	Other unintentional	Self-harm	Assault	Other intentional	Undetermined intent	Non injury	Total Injury
<1	-0.047	0.000	0.000	0.000	0.000	0.000	-0.001	0.000	-0.001	0.000	-0.001	-0.043	-0.004
1-4	-0.021	-0.003	0.000	-0.001	-0.001	-0.001	-0.001	0.000	-0.001	0.000	0.000	-0.014	-0.008
5-9	-0.009	-0.003	0.000	0.000	-0.001	-0.001	-0.001	0.000	0.000	0.000	0.000	-0.005	-0.005
10-14	-0.008	-0.005	0.000	0.000	0.000	0.000	0.000	0.000	0.000	0.000	0.000	-0.003	-0.005
15-19	-0.029	-0.021	0.000	0.000	0.000	0.000	0.000	-0.001	0.000	0.000	-0.001	-0.007	-0.023
20-24	-0.028	-0.015	0.000	-0.001	0.000	0.000	0.000	-0.002	-0.001	0.000	-0.001	-0.008	-0.020
25-29	-0.018	-0.008	0.000	0.000	0.000	0.000	0.000	-0.003	-0.001	0.000	-0.001	-0.006	-0.012
30-34	-0.008	-0.004	0.001	0.000	0.000	0.000	0.000	-0.001	0.000	0.000	0.000	-0.004	-0.004
35-39	-0.029	-0.005	0.001	0.000	0.000	0.000	0.000	-0.004	-0.001	0.000	-0.001	-0.019	-0.010
40-44	-0.064	-0.003	0.000	0.000	0.000	0.000	0.000	-0.004	0.000	0.000	-0.001	-0.054	-0.010
45-49	-0.087	-0.003	0.000	-0.001	0.000	0.000	0.000	-0.004	0.000	0.000	-0.001	-0.079	-0.008
50-54	-0.087	-0.002	0.000	0.000	0.000	0.000	0.000	-0.001	0.000	0.000	-0.001	-0.084	-0.003
55-59	-0.044	-0.001	0.000	0.000	0.000	0.000	0.001	-0.002	0.000	0.000	0.000	-0.041	-0.003
60-64	-0.009	-0.001	0.000	0.001	0.000	0.000	0.001	-0.002	0.000	0.000	0.000	-0.008	-0.001
65-69	-0.051	-0.001	0.000	0.000	0.000	0.000	0.001	-0.001	0.000	0.000	0.000	-0.050	-0.001
70-74	-0.079	-0.001	0.000	0.001	0.000	0.000	0.001	-0.001	0.000	0.000	0.000	-0.079	0.001
75-79	-0.067	0.000	0.000	0.001	0.000	0.000	0.001	0.000	0.000	0.000	0.000	-0.067	0.000
80-84	0.166	0.000	0.000	-0.002	0.000	0.000	-0.001	0.001	0.000	0.000	0.001	0.168	-0.002
≥85	0.348	0.001	0.000	-0.032	0.000	0.000	-0.016	0.003	0.000	0.000	0.004	0.389	-0.040
Total	**-0.170**	**-0.074**	**0.002**	**-0.033**	**-0.003**	**-0.002**	**-0.015**	**-0.021**	**-0.007**	**0.000**	**-0.004**	**-0.012**	**-0.158**

Table A.21: Contributions of injury deaths to changes in LD for females in Italy

Age groups	Age contribution	Transport	Poison	Fall	Fire	Drowning	Other unintentional	Self-harm	Assault	Other intentional	Undetermined intent	Non injury	Total Injury
<1	-0.066	0.000	0.000	0.000	0.000	0.000	-0.001	0.000	0.000	0.000	0.000	-0.065	-0.001
1-4	-0.015	-0.002	0.000	0.000	0.000	-0.001	-0.001	0.000	0.000	0.000	0.000	-0.011	-0.004
5-9	-0.009	-0.003	0.000	0.000	0.000	0.000	0.000	0.000	0.000	0.000	0.000	-0.006	-0.003
10-14	-0.005	-0.003	0.000	0.000	0.000	0.000	0.000	0.000	0.000	0.000	0.000	-0.003	-0.002
15-19	-0.020	-0.012	0.000	-0.001	0.000	0.000	-0.001	0.001	-0.001	0.000	0.000	-0.006	-0.013
20-24	-0.019	-0.011	0.000	0.000	0.000	0.000	-0.001	-0.001	-0.001	0.000	0.000	-0.006	-0.013
25-29	-0.018	-0.006	0.000	0.000	0.000	0.000	-0.001	-0.001	-0.001	0.000	0.000	-0.010	-0.008
30-34	-0.019	-0.004	0.000	0.000	0.000	0.000	0.000	-0.001	0.000	0.000	0.000	-0.014	-0.005
35-39	-0.024	-0.002	0.000	0.000	0.000	0.000	0.000	-0.001	-0.001	0.000	0.000	-0.020	-0.004
40-44	-0.040	-0.002	0.000	0.000	0.000	0.000	-0.001	0.000	0.000	0.000	0.000	-0.037	-0.003
45-49	-0.046	-0.002	0.000	0.000	0.000	0.000	0.000	0.000	0.000	0.000	0.000	-0.043	-0.003
50-54	-0.048	-0.001	0.000	0.000	0.000	0.000	0.000	0.000	0.000	0.000	0.000	-0.046	-0.002
55-59	-0.056	-0.002	0.000	0.000	0.000	0.000	0.000	-0.001	0.000	0.000	0.000	-0.053	-0.002
60-64	-0.047	-0.001	0.000	0.000	0.000	0.000	0.000	0.000	0.000	0.000	0.000	-0.046	-0.001
65-69	-0.074	-0.001	0.000	0.000	0.000	0.000	0.000	-0.001	0.000	0.000	0.000	-0.072	-0.003
70-74	-0.077	-0.001	0.000	0.000	0.000	0.000	0.000	0.000	0.000	0.000	0.000	-0.076	-0.001
75-79	-0.070	0.000	0.000	0.000	0.000	0.000	-0.001	0.000	0.000	0.000	0.000	-0.068	-0.002
80-84	0.088	0.000	0.000	0.000	0.000	0.000	0.002	0.000	0.000	0.000	0.000	0.085	0.002
≥85	0.533	0.001	0.000	-0.005	0.000	0.000	0.031	0.001	0.000	0.000	0.000	0.505	0.028
Total	**-0.033**	**-0.053**	**0.002**	**-0.006**	**0.000**	**-0.001**	**0.027**	**-0.005**	**-0.005**	**0.000**	**-0.001**	**0.008**	**-0.042**

Table A.22: Contributions of injury deaths to changes in LD for females in Japan

Age groups	Age contribution	Transport	Poison	Fall	Fire	Drowning	Other unintentional	Self-harm	Assault	Other intentional	Undetermined intent	Non injury	Total Injury
<1	-0.068	-0.001	0.000	0.000	0.000	0.000	-0.002	0.000	-0.001	0.000	0.000	-0.065	-0.004
1-4	-0.023	-0.003	0.000	-0.001	-0.001	-0.002	-0.001	0.000	-0.001	0.000	0.000	-0.013	-0.010
5-9	-0.014	-0.005	0.000	0.000	-0.001	-0.001	0.000	0.000	-0.001	0.000	0.000	-0.005	-0.009
10-14	-0.005	-0.002	0.000	0.000	0.000	0.000	0.000	0.002	0.000	0.000	0.000	-0.005	0.000
15-19	-0.015	-0.009	0.000	0.000	-0.001	0.000	0.000	0.003	0.000	0.000	0.000	-0.008	-0.007
20-24	-0.015	-0.008	0.000	0.000	-0.001	-0.001	0.000	0.003	0.000	0.000	0.000	-0.008	-0.007
25-29	-0.016	-0.003	0.000	-0.001	0.000	0.000	0.000	-0.002	0.000	0.000	0.000	-0.008	-0.007
30-34	-0.025	-0.002	0.000	-0.001	0.000	0.000	0.000	-0.006	-0.001	0.000	-0.001	-0.014	-0.011
35-39	-0.031	-0.002	0.000	0.000	0.000	0.000	0.000	-0.006	0.000	0.000	0.000	-0.021	-0.010
40-44	-0.033	-0.002	0.000	0.000	0.000	-0.001	0.000	-0.003	0.000	0.000	0.000	-0.027	-0.006
45-49	-0.035	-0.002	0.000	0.000	0.000	-0.001	0.000	-0.001	0.000	0.000	0.000	-0.030	-0.005
50-54	-0.056	-0.003	0.000	-0.001	0.000	-0.001	0.000	-0.003	0.000	0.000	0.000	-0.048	-0.008
55-59	-0.063	-0.003	0.000	-0.001	0.000	-0.001	0.000	-0.006	0.000	0.000	0.000	-0.051	-0.012
60-64	-0.071	-0.003	0.000	0.000	0.000	0.000	0.000	-0.006	0.000	0.000	0.000	-0.060	-0.011
65-69	-0.087	-0.003	0.000	0.000	0.000	0.000	0.000	-0.005	0.000	0.000	0.000	-0.077	-0.009
70-74	-0.089	-0.003	0.000	-0.001	0.000	0.000	0.000	-0.002	0.000	0.000	0.000	-0.083	-0.006
75-79	-0.057	-0.001	0.000	0.000	0.000	0.000	0.000	-0.001	0.000	0.000	0.000	-0.054	-0.003
80-84	0.080	0.001	0.000	0.000	0.000	0.000	0.001	0.001	0.000	0.000	0.000	0.077	0.003
≥85	0.374	0.006	0.000	-0.005	0.002	0.000	0.009	0.014	0.000	0.000	-0.001	0.350	0.024
Total	**-0.249**	**-0.051**	**0.000**	**-0.012**	**-0.005**	**-0.009**	**0.004**	**-0.018**	**-0.007**	**0.000**	**-0.002**	**-0.151**	**-0.098**

Table A.23: Contributions of injury deaths to changes in LD for females in the UK

Age groups	Age contribution	Transport	Poison	Fall	Fire	Drowning	Other unintentional	Self-harm	Assault	Other intentional	Undetermined intent	Non injury	Total Injury
<1	-0.083	0.000	0.000	0.000	0.000	0.000	0.000	0.000	0.000	0.000	-0.002	-0.080	-0.003
1-4	-0.026	-0.002	0.000	0.000	-0.002	-0.001	0.000	0.000	0.000	0.000	-0.002	-0.020	-0.006
5-9	-0.012	-0.002	0.000	0.000	0.000	0.000	0.001	0.000	0.000	0.000	-0.001	-0.009	-0.004
10-14	-0.013	-0.003	0.000	0.000	0.000	0.000	0.000	0.001	0.000	0.000	-0.001	-0.008	-0.005
15-19	-0.021	-0.010	0.001	0.000	0.000	0.000	0.001	0.005	0.000	0.000	-0.004	-0.014	-0.007
20-24	-0.024	-0.007	0.002	0.000	-0.001	0.000	0.001	0.002	0.000	0.000	-0.005	-0.016	-0.008
25-29	-0.016	-0.004	0.006	0.000	0.000	0.000	0.002	0.002	0.000	0.000	-0.005	-0.017	0.000
30-34	-0.013	-0.003	0.009	0.000	0.000	0.000	0.001	0.001	0.000	0.000	-0.004	-0.017	0.004
35-39	-0.008	-0.002	0.014	-0.001	0.000	0.000	0.001	0.001	0.000	0.000	-0.004	-0.017	0.009
40-44	-0.023	-0.002	0.013	0.000	-0.001	0.000	0.001	0.001	0.000	0.000	-0.002	-0.034	0.010
45-49	-0.046	-0.002	0.009	0.000	0.000	0.000	0.001	0.002	0.000	0.000	-0.002	-0.054	0.008
50-54	-0.060	-0.001	0.006	-0.001	0.000	0.000	0.001	0.001	0.000	0.000	-0.002	-0.065	0.005
55-59	-0.085	-0.001	0.003	0.000	0.000	0.000	0.001	0.000	0.000	0.000	-0.001	-0.087	0.003
60-64	-0.101	-0.001	0.002	0.000	0.000	0.000	0.001	0.000	0.000	0.000	-0.001	-0.103	0.002
65-69	-0.124	-0.001	0.001	0.001	0.000	0.000	0.001	0.000	0.000	0.000	0.000	-0.125	0.001
70-74	-0.119	0.000	0.000	0.001	0.000	0.000	0.000	0.000	0.000	0.000	0.000	-0.119	0.001
75-79	-0.041	0.000	0.000	0.000	0.000	0.000	0.000	0.000	0.000	0.000	0.000	-0.041	0.000
80-84	0.195	0.001	0.000	-0.003	0.000	0.000	0.002	0.000	0.000	0.000	0.000	0.195	0.000
≥85	0.462	0.001	0.000	-0.029	0.001	0.000	0.018	0.000	0.000	0.000	0.001	0.471	-0.009
Total	**-0.157**	**-0.038**	**0.065**	**-0.033**	**-0.005**	**-0.002**	**0.032**	**0.017**	**0.001**	**0.000**	**-0.035**	**-0.160**	**0.002**

Table A.24: Contributions of injury deaths to changes in LD for females in the US

Age groups	Age contribution	Transport	Poison	Fall	Fire	Drowning	Other unintentional	Self-harm	Assault	Other intentional	Undetermined intent	Non injury	Total Injury
<1	-0.068	-0.001	0.000	0.000	0.000	0.000	-0.001	0.000	0.000	0.000	0.000	-0.066	-0.003
1-4	-0.020	-0.003	0.000	0.000	-0.002	-0.001	0.000	0.000	-0.002	0.000	0.000	-0.012	-0.008
5-9	-0.009	-0.004	0.000	0.000	-0.001	0.000	0.000	0.000	0.000	0.000	0.000	-0.003	-0.005
10-14	-0.008	-0.006	0.000	0.000	0.000	0.000	0.000	0.004	0.000	0.000	0.000	-0.005	-0.003
15-19	-0.026	-0.025	0.002	0.000	0.000	0.000	0.000	0.007	-0.001	0.000	0.000	-0.008	-0.018
20-24	0.002	-0.010	0.018	0.000	-0.001	0.000	0.000	0.007	-0.002	0.000	0.000	-0.010	0.012
25-29	0.026	-0.003	0.030	0.000	0.000	0.000	0.000	0.005	-0.002	0.000	0.001	-0.005	0.031
30-34	0.035	-0.001	0.032	0.000	0.000	0.000	0.000	0.005	-0.001	0.000	0.001	0.000	0.035
35-39	0.012	-0.004	0.026	0.000	0.000	0.000	0.000	0.004	-0.001	0.000	-0.001	-0.012	0.024
40-44	-0.023	-0.003	0.016	0.000	0.000	0.000	0.000	0.002	-0.001	0.000	-0.001	-0.035	0.012
45-49	-0.030	-0.002	0.014	0.000	0.000	0.000	0.000	0.002	-0.001	0.000	0.000	-0.044	0.014
50-54	-0.018	-0.002	0.015	0.001	0.000	0.000	0.000	0.003	0.000	0.000	0.000	-0.036	0.017
55-59	-0.033	-0.001	0.011	0.001	0.000	0.000	0.000	0.002	0.000	0.000	0.001	-0.048	0.015
60-64	-0.072	-0.001	0.005	0.001	0.000	0.000	0.001	0.002	0.000	0.000	0.000	-0.078	0.007
65-69	-0.091	-0.001	0.001	0.001	0.000	0.000	0.000	0.001	0.000	0.000	0.000	-0.094	0.002
70-74	-0.057	-0.001	0.000	0.001	0.000	0.000	0.000	0.000	0.000	0.000	0.000	-0.058	0.001
75-79	-0.005	0.000	0.000	0.000	0.000	0.000	0.000	0.000	0.000	0.000	0.000	-0.005	0.000
80-84	0.162	0.001	0.000	-0.004	0.000	0.000	0.001	0.000	0.000	0.000	0.000	0.163	-0.001
≥85	0.393	0.001	0.000	-0.038	0.000	0.000	0.010	0.000	0.000	0.000	0.000	0.418	-0.026
Total	**0.169**	**-0.065**	**0.171**	**-0.038**	**-0.006**	**0.000**	**0.011**	**0.043**	**-0.011**	**0.000**	**0.001**	**0.063**	**0.106**

Table A.25: Contributions of injury deaths to changes in LD for females in all G7 countries combined

Age groups	Age contribution	Transport	Poison	Fall	Fire	Drowning	Other unintentional	Self-harm	Assault	Other intentional	Undetermined intent	Non injury	Total Injury
<1	-0.060	-0.001	0.000	0.000	0.000	0.000	-0.001	0.000	-0.001	0.000	0.000	-0.057	-0.003
1-4	-0.021	-0.003	0.000	0.000	-0.001	-0.001	-0.001	0.000	-0.001	0.000	0.000	-0.013	-0.008
5-9	-0.010	-0.004	0.000	0.000	-0.001	0.000	0.000	0.000	0.000	0.000	0.000	-0.005	-0.006
10-14	-0.008	-0.005	0.000	0.000	0.000	0.000	0.000	0.002	0.000	0.000	0.000	-0.005	-0.003
15-19	-0.023	-0.019	0.002	0.000	0.000	0.000	0.000	0.004	0.000	0.000	-0.001	-0.008	-0.015
20-24	-0.007	-0.010	0.010	0.000	0.000	0.000	0.000	0.003	-0.001	0.000	0.000	-0.009	0.002
25-29	0.011	-0.003	0.017	0.000	0.000	0.000	0.000	0.001	0.000	0.000	0.000	-0.004	0.015
30-34	0.013	-0.002	0.018	0.000	0.000	0.000	0.000	0.001	0.000	0.000	0.000	-0.003	0.016
35-39	-0.007	-0.003	0.015	0.000	0.000	0.000	0.000	0.000	-0.001	0.000	-0.001	-0.016	0.009
40-44	-0.040	-0.004	0.008	0.000	0.000	0.000	0.000	-0.001	-0.001	0.000	-0.001	-0.041	0.002
45-49	-0.049	-0.002	0.007	0.000	0.000	0.000	0.000	0.000	0.000	0.000	0.000	-0.052	0.003
50-54	-0.037	-0.002	0.008	0.000	0.000	0.000	0.000	0.000	0.000	0.000	0.000	-0.043	0.006
55-59	-0.032	-0.001	0.007	0.000	0.000	0.000	0.000	-0.001	0.000	0.000	0.000	-0.037	0.005
60-64	-0.038	-0.001	0.003	0.001	0.000	0.000	0.001	-0.001	0.000	0.000	0.000	-0.040	0.002
65-69	-0.075	-0.001	0.001	0.001	0.000	0.000	0.000	-0.001	0.000	0.000	0.000	-0.074	-0.001
70-74	-0.073	-0.001	0.000	0.001	0.000	0.000	0.000	0.000	0.000	0.000	0.000	-0.072	-0.001
75-79	-0.039	0.000	0.000	0.000	0.000	0.000	0.000	0.000	0.000	0.000	0.000	-0.039	0.000
80-84	0.152	0.001	0.000	-0.001	0.000	0.000	0.001	0.000	0.000	0.000	0.000	0.150	0.001
≥85	0.594	0.002	0.000	-0.025	0.001	-0.001	0.014	0.002	0.000	0.000	0.001	0.601	-0.007
Total	**0.250**	**-0.060**	**0.096**	**-0.026**	**-0.005**	**-0.003**	**0.015**	**0.010**	**-0.006**	**0.000**	**-0.003**	**0.232**	**0.018**

Table A.26: Contributions of injury deaths to changes in LD for males in Canada

Age groups	Age contribution	Transport	Poison	Fall	Fire	Drowning	Other unintentional	Self-harm	Assault	Other intentional	Undetermined intent	Non injury	Total Injury
<1	-0.048	-0.001	0.000	0.000	0.000	-0.001	-0.001	0.000	0.000	0.000	0.000	-0.046	-0.002
1-4	-0.018	-0.003	0.000	0.000	-0.002	-0.002	-0.001	0.000	-0.002	0.000	0.000	-0.009	-0.009
5-9	-0.013	-0.005	0.000	0.000	-0.001	-0.001	0.000	0.000	0.000	0.000	0.000	-0.006	-0.008
10-14	-0.015	-0.009	0.000	0.000	-0.001	-0.001	-0.001	0.000	0.000	0.000	0.000	-0.003	-0.012
15-19	-0.046	-0.040	0.008	-0.001	0.000	-0.001	-0.002	-0.002	0.001	0.000	-0.002	-0.007	-0.039
20-24	-0.015	-0.033	0.032	-0.002	-0.001	-0.001	-0.003	-0.002	-0.001	0.000	-0.002	-0.002	-0.013
25-29	0.036	-0.017	0.049	0.000	0.000	0.000	-0.002	0.001	0.001	0.000	-0.002	0.007	0.030
30-34	0.033	-0.007	0.046	0.000	-0.001	0.000	-0.001	-0.002	0.001	0.000	-0.002	-0.002	0.035
35-39	0.011	-0.009	0.041	0.000	-0.001	0.000	-0.001	-0.006	0.000	0.000	-0.003	-0.009	0.020
40-44	-0.025	-0.007	0.026	-0.001	-0.001	-0.001	-0.001	-0.005	0.000	0.000	-0.003	-0.031	0.006
45-49	-0.060	-0.007	0.022	-0.001	0.000	-0.001	-0.002	-0.002	0.001	0.000	-0.003	-0.067	0.008
50-54	-0.076	-0.004	0.018	0.000	0.000	0.000	0.000	-0.002	0.000	0.000	-0.002	-0.087	0.010
55-59	-0.097	-0.002	0.012	0.001	0.000	0.000	0.000	0.001	0.000	0.000	-0.001	-0.108	0.011
60-64	-0.119	-0.001	0.006	0.001	0.000	0.000	0.000	0.001	0.000	0.000	0.000	-0.126	0.007
65-69	-0.117	-0.001	0.002	0.001	0.000	0.000	-0.001	0.000	0.000	0.000	0.000	-0.118	0.000
70-74	-0.070	0.000	0.000	0.001	0.000	0.000	0.000	0.000	0.000	0.000	0.000	-0.070	0.000
75-79	0.047	0.000	0.000	-0.001	0.000	0.000	0.001	0.000	0.000	0.000	0.000	0.047	0.000
80-84	0.375	0.003	0.000	-0.006	0.000	0.000	0.004	0.000	0.000	0.000	0.000	0.374	0.001
≥85	0.600	0.003	0.000	-0.041	0.000	0.000	0.026	0.001	0.000	0.000	0.000	0.611	-0.011
Total	**0.383**	**-0.141**	**0.262**	**-0.051**	**-0.007**	**-0.008**	**0.015**	**-0.015**	**-0.001**	**0.001**	**-0.020**	**0.348**	**0.035**

Table A.27: Contributions of injury deaths to changes in LD for males in France

Age groups	Age contribution	Transport	Poison	Fall	Fire	Drowning	Other unintentional	Self-harm	Assault	Other intentional	Undetermined intent	Non injury	Total Injury
<1	-0.035	-0.001	0.000	0.000	0.000	0.000	-0.002	0.000	-0.001	0.000	0.000	-0.031	-0.004
1-4	-0.026	-0.004	0.000	-0.001	-0.001	-0.004	-0.002	0.000	0.000	0.000	0.000	-0.014	-0.012
5-9	-0.017	-0.004	0.000	0.000	-0.001	-0.001	-0.002	0.000	0.000	0.000	0.000	-0.008	-0.009
10-14	-0.021	-0.008	0.000	-0.001	0.000	-0.001	0.000	-0.001	-0.001	0.000	0.000	-0.009	-0.012
15-19	-0.079	-0.054	0.000	0.000	0.000	-0.001	-0.002	-0.007	0.000	0.000	0.000	-0.015	-0.064
20-24	-0.098	-0.061	-0.001	-0.001	-0.001	-0.002	-0.002	-0.015	0.000	0.000	0.002	-0.018	-0.081
25-29	-0.073	-0.038	0.000	-0.001	-0.001	-0.002	0.000	-0.017	0.000	0.000	0.002	-0.016	-0.057
30-34	-0.069	-0.022	0.001	-0.002	-0.001	-0.001	-0.001	-0.020	0.000	0.000	0.001	-0.023	-0.046
35-39	-0.091	-0.017	0.001	-0.002	-0.001	-0.002	-0.002	-0.019	-0.001	0.000	0.001	-0.050	-0.041
40-44	-0.143	-0.013	0.002	-0.003	-0.001	-0.002	-0.003	-0.020	0.000	0.000	0.001	-0.104	-0.038
45-49	-0.190	-0.010	0.002	-0.003	-0.001	-0.002	-0.002	-0.011	0.000	0.000	0.001	-0.164	-0.027
50-54	-0.173	-0.006	0.001	-0.002	0.000	-0.002	-0.001	-0.001	0.000	0.000	0.001	-0.161	-0.011
55-59	-0.149	-0.004	0.001	-0.001	0.000	-0.001	0.001	-0.001	0.000	0.000	0.001	-0.144	-0.004
60-64	-0.082	-0.003	0.001	-0.001	0.000	0.000	0.000	-0.001	0.000	0.000	0.001	-0.079	-0.003
65-69	-0.095	-0.002	0.000	0.000	0.000	0.000	0.000	-0.002	0.000	0.000	0.000	-0.091	-0.004
70-74	-0.057	-0.001	0.000	0.000	0.000	0.000	0.000	-0.001	0.000	0.000	0.000	-0.055	-0.002
75-79	0.058	0.001	0.000	0.000	0.000	0.000	0.001	0.001	0.000	0.000	0.000	0.056	0.002
80-84	0.420	0.003	0.000	0.000	0.000	0.000	0.008	0.004	0.000	0.000	0.000	0.405	0.015
≥85	0.568	0.003	-0.001	-0.011	0.000	0.001	0.026	0.008	0.000	0.000	-0.001	0.543	0.025
Total	**-0.352**	**-0.242**	**0.007**	**-0.029**	**-0.006**	**-0.021**	**0.016**	**-0.104**	**-0.003**	**0.000**	**0.009**	**0.022**	**-0.374**

Table A.28: Contributions of injury deaths to changes in LD for males in Germany

Age groups	Age contribution	Transport	Poison	Fall	Fire	Drowning	Other unintentional	Self-harm	Assault	Other intentional	Undetermined intent	Non injury	Total Injury
<1	-0.055	0.000	0.000	0.000	0.000	0.000	-0.001	0.000	-0.001	0.000	-0.001	-0.052	-0.003
1-4	-0.021	-0.003	0.000	0.000	-0.002	-0.003	0.000	0.000	0.000	0.000	-0.001	-0.012	-0.009
5-9	-0.011	-0.004	0.000	0.000	-0.001	-0.001	0.000	0.000	0.000	0.000	0.000	-0.005	-0.006
10-14	-0.016	-0.005	0.000	0.000	0.000	-0.001	-0.001	-0.001	0.000	0.000	-0.001	-0.007	-0.009
15-19	-0.071	-0.047	0.000	-0.001	0.000	0.000	0.000	-0.008	-0.001	0.000	-0.002	-0.011	-0.059
20-24	-0.089	-0.051	-0.003	-0.001	0.000	0.000	0.000	-0.011	-0.001	0.000	-0.002	-0.018	-0.071
25-29	-0.057	-0.022	-0.003	-0.001	0.000	0.001	-0.001	-0.009	-0.001	0.000	-0.002	-0.017	-0.040
30-34	-0.037	-0.012	0.001	-0.001	0.000	0.000	-0.001	-0.008	-0.001	0.000	-0.001	-0.014	-0.023
35-39	-0.056	-0.011	0.002	-0.001	0.000	0.000	-0.001	-0.010	-0.001	0.000	-0.001	-0.033	-0.023
40-44	-0.104	-0.009	0.002	-0.001	-0.001	0.000	0.000	-0.012	0.000	0.000	-0.001	-0.082	-0.023
45-49	-0.156	-0.006	0.001	-0.002	0.000	0.000	-0.001	-0.010	-0.001	0.000	-0.002	-0.134	-0.022
50-54	-0.144	-0.003	0.001	-0.001	0.000	0.000	0.000	-0.005	-0.001	0.000	-0.001	-0.133	-0.011
55-59	-0.097	-0.001	0.001	0.000	0.000	0.000	0.001	-0.002	0.000	0.000	0.000	-0.095	-0.002
60-64	-0.064	-0.001	0.000	0.001	0.000	0.000	0.002	-0.001	0.000	0.000	0.000	-0.065	0.000
65-69	-0.081	-0.001	0.000	0.001	0.000	0.000	0.001	-0.001	0.000	0.000	0.000	-0.081	0.000
70-74	-0.038	0.000	0.000	0.000	0.000	0.000	0.000	0.000	0.000	0.000	0.000	-0.038	0.000
75-79	0.090	0.000	0.000	-0.001	0.000	0.000	-0.001	0.001	0.000	0.000	0.000	0.091	-0.001
80-84	0.391	0.001	0.000	-0.007	0.000	0.000	-0.004	0.003	0.000	0.000	0.001	0.396	-0.005
≥85	0.352	0.001	0.000	-0.022	0.000	0.000	-0.009	0.004	0.000	0.000	0.001	0.378	-0.025
Total	**-0.265**	**-0.175**	**0.003**	**-0.039**	**-0.006**	**-0.007**	**-0.013**	**-0.072**	**-0.008**	**0.000**	**-0.015**	**0.067**	**-0.331**

Table A.29: Contributions of injury deaths to changes in LD for males in Italy

Age groups	Age contribution	Transport	Poison	Fall	Fire	Drowning	Other unintentional	Self-harm	Assault	Other intentional	Undetermined intent	Non injury	Total Injury
<1	-0.064	0.000	0.000	0.000	0.000	0.000	-0.001	0.000	0.000	0.000	0.000	-0.062	-0.002
1-4	-0.011	-0.002	0.000	-0.001	0.000	0.000	0.000	0.000	0.001	0.000	0.000	-0.008	-0.003
5-9	-0.010	-0.002	0.000	0.000	0.000	0.000	-0.001	0.000	0.000	0.000	0.000	-0.007	-0.003
10-14	-0.015	-0.008	0.000	0.000	0.000	0.000	-0.001	0.000	0.000	0.000	0.000	-0.005	-0.009
15-19	-0.060	-0.043	0.000	-0.001	0.000	0.002	-0.003	0.000	-0.002	0.000	0.000	-0.012	-0.048
20-24	-0.082	-0.057	0.001	-0.001	0.000	0.000	-0.006	0.000	-0.004	0.000	0.000	-0.013	-0.069
25-29	-0.072	-0.041	0.001	-0.002	0.000	0.000	-0.004	-0.004	-0.004	0.000	0.000	-0.018	-0.054
30-34	-0.056	-0.024	0.001	-0.001	0.000	0.000	-0.003	-0.002	-0.003	0.000	0.000	-0.024	-0.032
35-39	-0.062	-0.013	0.002	-0.001	0.000	-0.001	-0.003	-0.002	-0.003	0.000	0.000	-0.042	-0.020
40-44	-0.069	-0.009	0.002	-0.001	0.000	-0.001	-0.001	-0.002	-0.002	0.000	0.000	-0.055	-0.014
45-49	-0.076	-0.007	0.002	-0.001	0.000	0.000	-0.001	-0.001	-0.001	0.000	0.000	-0.066	-0.010
50-54	-0.091	-0.004	0.001	-0.001	0.000	0.000	0.000	0.001	-0.001	0.000	0.000	-0.087	-0.004
55-59	-0.113	-0.002	0.000	-0.001	0.000	0.000	0.001	0.001	0.000	0.000	0.000	-0.111	-0.002
60-64	-0.122	-0.002	0.000	-0.001	0.000	0.000	0.000	0.000	0.000	0.000	0.000	-0.119	-0.002
65-69	-0.127	-0.001	0.000	0.000	0.000	0.000	0.000	0.000	0.000	0.000	0.000	-0.125	-0.002
70-74	-0.089	-0.001	0.000	0.000	0.000	0.000	0.000	-0.001	0.000	0.000	0.000	-0.088	-0.002
75-79	0.013	0.000	0.000	0.000	0.000	0.000	0.000	0.000	0.000	0.000	0.000	0.013	0.000
80-84	0.296	0.002	0.000	-0.001	0.000	0.000	0.003	0.002	0.000	0.000	0.000	0.290	0.006
≥85	0.399	0.002	0.000	-0.005	0.000	0.000	0.007	0.002	0.000	0.000	0.000	0.394	0.005
Total	**-0.411**	**-0.212**	**0.009**	**-0.018**	**-0.001**	**-0.002**	**-0.013**	**-0.006**	**-0.020**	**0.000**	**-0.002**	**-0.147**	**-0.263**

Table A.30: Contributions of injury deaths to changes in LD for males in Japan

Age groups	Age contribution	Transport	Poison	Fall	Fire	Drowning	Other unintentional	Self-harm	Assault	Other intentional	Undetermined intent	Non injury	Total Injury
<1	-0.069	0.000	0.000	-0.001	0.000	0.000	-0.003	0.000	-0.001	0.000	0.001	-0.065	-0.004
1-4	-0.029	-0.005	0.000	-0.001	-0.001	-0.004	-0.001	0.000	-0.002	0.000	0.000	-0.014	-0.015
5-9	-0.016	-0.006	0.000	0.000	-0.001	-0.002	0.000	0.000	-0.001	0.000	0.000	-0.005	-0.011
10-14	-0.007	-0.004	0.000	0.000	0.000	-0.001	0.000	0.003	0.000	0.000	0.001	-0.005	-0.002
15-19	-0.041	-0.032	0.000	-0.001	-0.001	-0.001	0.000	0.008	-0.001	0.000	0.000	-0.015	-0.027
20-24	-0.031	-0.025	0.000	-0.002	0.000	-0.002	-0.001	0.009	-0.001	0.000	0.001	-0.011	-0.020
25-29	-0.038	-0.017	-0.001	-0.002	-0.001	-0.001	-0.001	-0.003	-0.001	0.000	-0.001	-0.012	-0.026
30-34	-0.046	-0.013	-0.001	-0.002	-0.001	-0.001	-0.001	-0.007	-0.001	0.000	-0.001	-0.021	-0.026
35-39	-0.063	-0.009	0.000	-0.001	-0.001	-0.001	-0.001	-0.014	-0.001	0.000	-0.001	-0.034	-0.029
40-44	-0.087	-0.008	-0.001	-0.001	-0.001	-0.002	-0.001	-0.025	-0.001	0.000	-0.001	-0.049	-0.039
45-49	-0.100	-0.006	0.000	-0.002	-0.001	-0.002	-0.001	-0.023	0.000	0.000	-0.001	-0.064	-0.036
50-54	-0.139	-0.006	0.000	-0.002	-0.001	-0.001	-0.001	-0.026	-0.001	0.000	-0.001	-0.100	-0.039
55-59	-0.154	-0.006	0.000	-0.002	-0.001	-0.001	0.000	-0.026	0.000	0.000	-0.001	-0.117	-0.037
60-64	-0.121	-0.005	0.000	-0.002	0.000	-0.001	0.000	-0.014	0.000	0.000	0.000	-0.098	-0.023
65-69	-0.110	-0.003	0.000	-0.001	0.000	0.000	0.000	-0.007	0.000	0.000	0.000	-0.098	-0.012
70-74	-0.072	-0.002	0.000	-0.001	0.000	0.000	0.000	-0.001	0.000	0.000	0.000	-0.068	-0.004
75-79	0.018	0.000	0.000	0.000	0.000	0.000	0.000	0.000	0.000	0.000	0.000	0.017	0.001
80-84	0.245	0.004	0.000	0.000	0.001	0.000	0.003	0.003	0.000	0.000	0.000	0.235	0.010
≥85	0.323	0.006	0.000	-0.006	0.002	-0.001	0.004	0.007	0.000	0.000	-0.001	0.312	0.012
Total	**-0.539**	**-0.136**	**-0.003**	**-0.025**	**-0.008**	**-0.021**	**-0.006**	**-0.115**	**-0.010**	**0.000**	**-0.004**	**-0.211**	**-0.328**

Table A.31: Contributions of injury deaths to changes in LD for males in the UK

Age groups	Age contribution	Transport	Poison	Fall	Fire	Drowning	Other unintentional	Self-harm	Assault	Other intentional	Undetermined intent	Non injury	Total Injury
<1	-0.097	0.000	0.000	0.000	0.000	0.000	0.001	0.000	0.000	0.000	-0.002	-0.094	-0.003
1-4	-0.029	-0.003	0.000	0.000	-0.002	-0.001	0.001	0.000	0.000	0.000	-0.002	-0.021	-0.008
5-9	-0.013	-0.003	0.000	0.000	-0.001	-0.001	0.000	0.000	0.000	0.000	-0.001	-0.008	-0.006
10-14	-0.018	-0.007	0.000	-0.001	0.000	0.000	0.001	0.001	0.000	0.000	-0.001	-0.011	-0.007
15-19	-0.061	-0.032	0.002	0.000	-0.001	0.000	0.003	0.003	0.000	0.000	-0.011	-0.024	-0.037
20-24	-0.077	-0.030	0.005	-0.002	-0.001	0.000	0.004	0.000	0.000	0.000	-0.016	-0.038	-0.039
25-29	-0.056	-0.019	0.011	-0.001	-0.001	0.000	0.003	-0.002	0.000	0.000	-0.013	-0.034	-0.022
30-34	-0.044	-0.014	0.022	-0.001	-0.001	0.000	0.002	-0.002	0.001	0.000	-0.012	-0.038	-0.006
35-39	-0.021	-0.010	0.029	-0.001	-0.001	0.000	0.001	-0.001	0.000	0.000	-0.009	-0.032	0.010
40-44	-0.024	-0.006	0.032	-0.001	-0.001	0.000	0.002	0.002	0.000	0.000	-0.005	-0.046	0.023
45-49	-0.046	-0.004	0.023	-0.001	-0.001	0.000	0.002	0.005	0.000	0.000	-0.004	-0.066	0.020
50-54	-0.071	-0.002	0.012	-0.001	0.000	0.000	0.001	0.005	0.000	0.000	-0.003	-0.084	0.013
55-59	-0.117	-0.002	0.005	0.000	0.000	0.000	0.001	0.002	0.000	0.000	-0.002	-0.122	0.005
60-64	-0.145	-0.001	0.002	0.000	0.000	0.000	0.002	0.001	0.000	0.000	-0.001	-0.148	0.003
65-69	-0.135	-0.001	0.000	0.001	0.000	0.000	0.001	0.000	0.000	0.000	0.000	-0.136	0.001
70-74	-0.070	0.000	0.000	0.000	0.000	0.000	0.000	0.000	0.000	0.000	0.000	-0.070	0.000
75-79	0.085	0.000	0.000	-0.001	0.000	0.000	0.000	0.000	0.000	0.000	0.000	0.085	0.000
80-84	0.440	0.001	0.000	-0.005	0.000	0.000	0.001	0.000	0.000	0.000	0.000	0.442	-0.002
≥85	0.466	0.002	0.000	-0.021	0.000	0.000	0.004	0.000	0.000	0.000	0.000	0.481	-0.015
Total	**-0.034**	**-0.131**	**0.143**	**-0.036**	**-0.009**	**-0.002**	**0.029**	**0.016**	**0.001**	**0.000**	**-0.080**	**0.035**	**-0.070**

Table A.32: Contributions of injury deaths to changes in LD for males in the US

Age groups	Age contribution	Transport	Poison	Fall	Fire	Drowning	Other unintentional	Self-harm	Assault	Other intentional	Undetermined intent	Non injury	Total Injury
<1	-0.078	-0.001	0.000	0.000	0.000	0.000	-0.001	0.000	-0.001	0.000	0.000	-0.076	-0.002
1-4	-0.022	-0.005	0.000	-0.001	-0.003	-0.001	-0.001	0.000	-0.002	0.000	0.001	-0.011	-0.011
5-9	-0.011	-0.006	0.000	0.000	-0.001	0.000	0.000	0.000	0.000	0.000	0.000	-0.004	-0.007
10-14	-0.013	-0.008	0.000	0.000	-0.001	-0.001	-0.001	0.004	0.000	0.000	0.000	-0.006	-0.007
15-19	-0.051	-0.045	0.002	-0.001	-0.001	-0.002	-0.001	0.011	-0.002	0.000	0.000	-0.012	-0.039
20-24	-0.022	-0.034	0.031	-0.001	-0.001	-0.002	-0.002	0.013	-0.013	0.000	-0.001	-0.013	-0.009
25-29	0.053	-0.010	0.061	-0.001	-0.001	-0.001	-0.001	0.012	-0.003	0.000	0.001	-0.006	0.059
30-34	0.065	-0.005	0.065	0.000	-0.001	0.000	-0.001	0.010	0.003	0.000	0.001	-0.008	0.073
35-39	0.031	-0.004	0.052	0.000	-0.001	0.000	0.000	0.008	0.002	0.000	-0.001	-0.025	0.056
40-44	-0.030	-0.006	0.032	-0.001	-0.001	0.000	-0.001	0.004	0.001	0.000	-0.001	-0.058	0.028
45-49	-0.064	-0.004	0.023	0.000	-0.001	0.000	-0.001	0.004	0.000	0.000	-0.001	-0.085	0.021
50-54	-0.043	-0.001	0.022	0.001	0.000	0.000	0.000	0.004	0.000	0.000	0.000	-0.070	0.027
55-59	-0.028	0.001	0.016	0.001	0.000	0.000	0.001	0.004	0.000	0.000	0.001	-0.051	0.024
60-64	-0.043	0.000	0.007	0.001	0.000	0.000	0.001	0.002	0.000	0.000	0.000	-0.055	0.012
65-69	-0.050	0.000	0.002	0.001	0.000	0.000	0.000	0.001	0.000	0.000	0.000	-0.054	0.003
70-74	-0.005	0.000	0.000	0.000	0.000	0.000	0.000	0.000	0.000	0.000	0.000	-0.005	0.000
75-79	0.084	0.000	0.000	-0.002	0.000	0.000	0.000	0.000	0.000	0.000	0.000	0.085	-0.001
80-84	0.345	0.002	0.000	-0.006	0.000	0.000	0.002	0.001	0.000	0.000	0.000	0.346	0.000
≥85	0.409	0.003	0.000	-0.020	0.000	0.000	0.007	0.001	0.000	0.000	0.000	0.418	-0.009
Total	**0.526**	**-0.123**	**0.313**	**-0.028**	**-0.009**	**-0.007**	**0.002**	**0.079**	**-0.014**	**0.002**	**0.001**	**0.309**	**0.217**

Table A.33: Contributions of injury deaths to changes in LD for males in all G7 countries combined

Age groups	Age contribution	Transport	Poison	Fall	Fire	Drowning	Other unintentional	Self-harm	Assault	Other intentional	Undetermined intent	Non injury	Total Injury
<1	-0.067	-0.001	0.000	0.000	0.000	0.000	-0.001	0.000	-0.001	0.000	0.000	-0.064	-0.003
1-4	-0.023	-0.004	0.000	-0.001	-0.002	-0.002	-0.001	0.000	-0.001	0.000	0.000	-0.013	-0.010
5-9	-0.013	-0.005	0.000	0.000	-0.001	-0.001	0.000	0.000	0.000	0.000	0.000	-0.005	-0.007
10-14	-0.013	-0.007	0.000	0.000	0.000	-0.001	0.000	0.002	0.000	0.000	0.000	-0.006	-0.007
15-19	-0.053	-0.042	0.002	-0.001	-0.001	-0.001	-0.001	0.006	0.000	0.000	-0.001	-0.013	-0.040
20-24	-0.037	-0.036	0.018	-0.001	-0.001	-0.001	-0.001	0.006	-0.005	0.000	-0.001	-0.014	-0.023
25-29	0.014	-0.015	0.036	-0.001	-0.001	0.000	-0.001	0.003	0.001	0.000	-0.001	-0.009	0.022
30-34	0.017	-0.009	0.037	-0.001	-0.001	0.000	-0.001	0.001	0.002	0.000	-0.001	-0.012	0.030
35-39	-0.010	-0.008	0.030	-0.001	-0.001	0.000	-0.001	-0.001	0.001	0.000	-0.001	-0.029	0.019
40-44	-0.065	-0.008	0.018	-0.001	-0.001	-0.001	-0.001	-0.005	0.000	0.000	-0.001	-0.065	0.000
45-49	-0.095	-0.006	0.013	-0.001	-0.001	-0.001	-0.001	-0.004	-0.001	0.000	-0.001	-0.094	-0.001
50-54	-0.084	-0.003	0.012	0.000	0.000	0.000	0.000	-0.004	0.000	0.000	0.000	-0.088	0.004
55-59	-0.069	-0.001	0.009	0.000	0.000	0.000	0.001	-0.003	0.000	0.000	0.000	-0.075	0.007
60-64	-0.065	-0.001	0.005	0.000	0.000	0.000	0.001	-0.001	0.000	0.000	0.000	-0.068	0.003
65-69	-0.080	-0.001	0.001	0.000	0.000	0.000	0.000	-0.001	0.000	0.000	0.000	-0.079	0.000
70-74	-0.039	0.000	0.000	0.000	0.000	0.000	0.000	0.000	0.000	0.000	0.000	-0.038	0.000
75-79	0.067	0.001	0.000	0.000	0.000	0.000	0.000	0.000	0.000	0.000	0.000	0.067	0.000
80-84	0.349	0.003	0.000	-0.003	0.000	0.000	0.002	0.002	0.000	0.000	0.000	0.346	0.003
≥85	0.425	0.003	0.000	-0.016	0.001	0.000	0.005	0.003	0.000	0.000	0.000	0.430	-0.005
Total	**0.160**	**-0.141**	**0.182**	**-0.027**	**-0.008**	**-0.010**	**0.001**	**0.003**	**-0.002**	**0.001**	**-0.008**	**0.168**	**-0.008**
